# Loss of ST8Sia6 mediated α-2,8-linked di-sialylation enhances T cell-dependent antibody responses and promotes autoimmunity in aged mice

**DOI:** 10.3389/fimmu.2026.1874115

**Published:** 2026-07-02

**Authors:** Ronja Brüchert, Michael Hinzpeter-Schmidt, Bettina Röder, Mareike Krause, Stefanie Brey, Thomas H. Winkler, Falk F. R. Buettner, Martina Mühlenhoff, Lars Nitschke

**Affiliations:** 1 Division of Genetics, Department of Biology, FAU University of Erlangen-Nürnberg, Erlangen, Germany; 2 Institute of Clinical Biochemistry, Hannover Medical School, Hannover, Germany; 3 Proteomics, Institute of Theoretical Medicine, Faculty of Medicine, University of Augsburg, Augsburg, Germany

**Keywords:** autoimmune disease, B cells, CD45, plasma cells, sialoglycan, thymus-dependent immune response

## Abstract

**Introduction:**

Sialic acids are terminal nine-carbon sugars that form sialoglycans on the glycocalyx of all vertebrate cell surfaces. In this exposed position, they fulfill various functions in the immune system, including involvement in host-pathogen interactions and control of immune cell activation. The synthesis of sialoglycans is catalyzed by sialyltransferases. One of these is ST8Sia6, which is known to catalyze the synthesis of α-2,8-linked di-sialyl motifs and to be upregulated in plasma cells.

**Methods:**

We studied the role of ST8Sia6 in the immune system by examining a *St8sia6* knockout mouse model.

**Results:**

The *St8sia6^-/-^* mice showed normal B cell and plasma cell development. However, in *St8sia6^-/-^* B cells, a stronger proliferation was induced by T cell-dependent stimuli. Additionally, an increased IgG1 response was observed upon T cell-dependent immunization. In the study, 60-week-old *St8sia6^-/-^* mice showed enlarged populations of germinal center B cells, age-induced B cells, and plasma cells. Furthermore, aged *St8sia6^-/-^* mice exhibited increased activation markers on their B cells and displayed elevated levels of anti-nuclear IgG autoantibodies, all of which are indicative of autoimmunity. We identified CD45 as a carrier protein of *O*-acetylated α2,8-linked di-sialic acids on plasma cells.

**Discussion:**

These results demonstrate that the loss of ST8Sia6-mediated α-2,8-linked di-sialylation enhances T cell-dependent immune responses and promotes an accumulation of strongly activated B cells and an autoimmune-like phenotype upon aging.

## 1 Introduction

Cell surface glycosylation is a common post-translational modification that has the ability to modulate immune cell interactions (1). The termini of the glycan chains are frequently capped with the negatively charged, acidic nine-carbon sugar sialic acid (Sia) (2). Sias can be attached to N- and O-glycans of secreted and transmembrane glycoproteins and to the glycan moiety of glycolipids (3, 4). Due to their exposed position, Sias have diverse functions in several biological processes, including cell-cell interactions, cell migration, control of the complement system, and host-pathogen interactions (5). Moreover, Sias are ligands for sialic acid-binding immunoglobulin-like lectins (Siglecs). As such, they play a crucial role in immune cell signaling and in self/non-self discrimination through binding to Siglecs (6–8). Commonly, Sias are attached to their underlying monosaccharide on lymphocytes via α-2,3- or α-2,6-linkage to galactose (Gal) or *N*-acetylgalactosamine (GalNAc), resulting in mono-sialylated Sia-α-2,3/6-Gal and Sia-α-2,6-GalNAc structures (9). Moreover, Sia can be attached to another Sia residue through an α-2,8-linkage (Sia-α-2,8-Sia), leading to di-, oligo-, and poly-sialylated structures (diSia, oligoSia, and polySia, respectively) (10). The biosynthesis of sialylated glycans (sialoglycans) occurs in the Golgi apparatus and is catalyzed by a family of 20 linkage- and acceptor-specific sialyltransferases (STs). STs transfer Sia from cytosine-5`-monophosphate (CMP)-activated Sia to Gal, GalNAc, or Sia (10–12). Sia-α-2,8-Sia linkages are formed by the six members of the ST8Sia subfamily (ST8Sia1-6). One of these members, ST8Sia6, generates α-2,8-linked diSia preferentially on O-glycans (12). Wang et al. demonstrated that the expression of *St8sia6* significantly increased during B cell activation and differentiation into plasma cells, a process that coincided with increased levels of diSia groups (13). In these experiments, primary B cells were stimulated *in vitro* in a T cell-dependent (TD) manner (using IL-4/IL-5 and CD40L) and a T cell-independent (TI) manner (using lipopolysaccharide (LPS)). *St8sia6* expression was significantly increased in plasma cells by both types of stimulation (13).

Plasma cells are antibody-secreting cells that are terminally differentiated B cells. Naïve B cells develop in the bone marrow and then migrate to secondary lymphoid organs (such as the spleen and lymph nodes), and in the spleen, they undergo further maturation processes and become follicular and marginal zone B cells (14). After antigen encounter, two types of immune responses may occur, either in a TD- or TI-dependent manner, depending on the antigen (15, 16). Upon stimulation, B cells become activated and upregulate early activation markers such as MHC-II and CD86. In the case of TD stimulation, activated B cells internalize the antigen and present it on MHC-II to helper T cells, which induces clonal expansion of B cells and formation of germinal centers in lymph nodes or the spleen. Within germinal centers, B cells undergo hypermutation of their B cell receptor, and immunoglobulin (Ig) isotype class-switching is induced. These processes take place with the support of CD4^+^ T follicular helper cells. They control the selection of germinal center (GC) B cells with high-affinity B cell receptors and maintain the germinal center through the induction of further clonal expansion (17, 18). After affinity maturation and selection of GC B cells, positively selected B cells leave the germinal center and become antibody-secreting plasmablasts, which develop into long-lived plasma cells secreting mainly IgG (19, 20). Following an encounter with TI-antigens, which are typically antigens with repetitive epitopes, B cells also proliferate but do not enter germinal centers, instead directly differentiating into plasmablasts and short-lived plasma cells that secrete mainly IgM (16). Impaired negative selection in the bone marrow or impaired selection in the GC can lead to B cells with B cell receptors (BCRs) directed against self-structures that differentiate into autoantibody-secreting plasmablasts and plasma cells. These autoantibodies play major roles in several autoimmune diseases, including rheumatoid arthritis and systemic lupus erythematosus (SLE) (21, 22). In these diseases, autoantibodies against self-structures are formed, causing severe inflammation and organ damage (23, 24). In general, autoimmune diseases occur when the immune system is no longer able to discriminate between self and non-self structures. Major structures that support the immune system in this decision are surface glycosylation sialoglycans (25). There are indications that sialoglycans are involved in preventing autoimmunity. For example, the gene *St8sia4*, coding for a poly-Sia-generating enzyme, was detected as a locus linked to SLE in genome-wide association studies (26). It was shown that overexpression of *St8sia6* led to tolerance in inflammation and autoimmunity in an induced type 1 diabetes mouse model (6), but otherwise the link of this gene to autoimmunity is not known. Some tumors also alter their surface glycosylation in order to escape immune responses (27). Enhanced growth of tumor cell lines was seen upon *St8sia6* overexpression, leading to decreased survival of the mice due to inhibition of anti-tumor immune responses (28).

Specific sialoglycans serve as ligands for Siglecs. The α-2,8-di-sialyl motif catalyzed by ST8Sia6 is known to generate ligands for murine Siglec-E and human Siglec-7 and Siglec-9 (6, 29, 30). Siglec-E is a CD33-related Siglec that is mainly expressed on neutrophils, splenic dendritic cells, mature NK cells, and macrophages (31). Siglecs have the ability to bind their ligands either *in cis* (on the same cell) or *in trans* (on a different cell) (32). The binding between Siglecs and sialoglycans depends on several factors, including the glycosidic linkage type of Sia, the nature of the penultimate sugar, and the composition of the underlying glycan structure (33). Moreover, variations in the Sia type, which arise from different modifications, can significantly alter Siglec binding by changing the chemical properties and steric accessibility of Sia. One of the most prevalent Sia modifications is 9-*O*-acetylation, which occurs as 9-mono- and 7,9-di-*O*-acetylation (34, 35). This modification is catalyzed by the sialate *O*-acetyltransferase Cas1 domain-containing 1 (CASD1). CASD1 is a Golgi-resident enzyme that transfers *O*-acetyl groups onto CMP-Sia, the donor substrate of the 20 STs (3, 36). Notably, recent studies in CHO cells identified ST8Sia6 as a key driver for the formation of 9-*O*- and 7,9-*O*-acetylated sialoglycans (36). The generated *O*-acetylated α-2,8-linked Sia residues can be detected by virolectins, which are derived from the hemagglutinin-esterase (HE) proteins of influenza C virus (ICV) and bovine coronavirus (BCoV). These viral probes, which encompass the ectodomain of HE variants with an inactive esterase domain (HE^0^), enable the detection of 9-*O*-acetylated (ICV-HE^0^) and 7,9-*O*-acetylated (BCoV-HE^0^) sialoglycans (3).

Knowing that ST8Sia6 is significantly upregulated in plasma cells, we hypothesized that this sialyltransferase is required for plasma cell functions and could potentially also affect B cell tolerance. To investigate the biological relevance of ST8Sia6-mediated α-2,8-di-sialylation on plasma cells, we established an *St8sia6* knockout mouse model. Here, we demonstrate that all B cell populations are normally formed and that early B cell activation is also unaltered in *St8sia6*-deficient mice. Notably, upon T cell-dependent stimulation, we observed increased B cell proliferation *in vitro* and higher antigen-specific Ig responses in *St8sia6^-/-^* mice. Additionally, aged *St8Sia6^-/-^* mice develop a highly activated B cell phenotype with autoimmune-like characteristics.

## 2 Methods

### 2.1 Mice

The *St8sia6*^-/-^ mouse used in this study was established using CRISPR/Cas9 technology. For this, two guide RNAs (5´-GACTTAACAGATACAGCGAG-3´ and 3´-GGTGGCCTGTCAGAGAACGA-5´) flanking exon 5 of *St8sia6* were used to delete exon 5 (**Supplementary Figure 1**). Aberrant splicing of *St8sia6* would create a premature stop codon in exon 6, leading to a deletion of the enzyme ST8Sia6 in these mice. These mice were created on a C57BL/6 background, crossed back several times with C57BL/6 mice to eliminate potential off-target mutations, and subsequently a *St8sia6*^-/-^ colony and a *St8sia6*^+/+^ control colony were established from littermates for all further experiments. *Casd1*^fl/fl^ mice were generated by Martina Mühlenhoff (unpublished results) and crossed with Mb1Cre^+/-^ (kindly provided by Elias Hobeika, Ulm University Hospital) and LckCre^+/-^ (purchased from the Jackson Laboratory, Maine, USA) mice. The *Casd1* conditional knockout mice were also generated on a C57BL/6 background. Both male and female mice were included in all experiments, with the exception of autoimmune analyses (anti-nuclear antibodies (ANA), blood urea nitrogen (BUN), and anti-dsDNA ELISA), which were performed exclusively in female mice. Experiments were performed in accordance with German animal welfare law and were approved by the animal welfare committee.

### 2.2 Immunization

To induce a thymus-dependent immune response, *St8Sia6*^-/-^ and *St8Sia6*^+/+^ mice were immunized intraperitoneally with 100 µl 4-hydroxy-3-nitrophenylacetyl hapten–keyhole limpet hemocyanin (NP-KLH; Biosearch Technologies, Petaluma, USA) in aluminum hydroxide (SERVA Electrophoresis GmbH, Heidelberg, Germany) as an adjuvant. Specifically, 20 µl NP-KLH with a concentration of 1 mg/mL was mixed with 20 µl aluminum hydroxide and 60 µl PBS for 45 min at room temperature in the dark. To induce a thymus-independent immune response, *St8Sia6*^-/-^ and *St8Sia6*^+/+^ mice were immunized intraperitoneally with 100µl PBS with 10 µg NP-Ficoll (Creative Diagnostics, New York City, USA). Blood was collected from the tail vein on days 0, 7, 14, 21, and 28 for NP-KLH and on days 0, 5, 7, 10, and 14 for NP-Ficoll. Mice were analyzed 14 or 28 days after immunization.

### 2.3 Cell preparation and flow cytometry

Single-cell suspensions of bone marrow, spleen, lymph nodes, and thymus were prepared in PBS (Gibco, Waltham, USA). For bone marrow, spleen, and thymus cell suspensions, erythrocytes were depleted using 1X ACK lysis buffer. Subsequently, the cells were washed and stained with F_c_-block (2.4G2, hybridoma generated in-house) to avoid cross-linking of antibodies and non-specific binding. During this pre-step, a fixable viability dye (eBio, Waltham, USA) was used to stain dead cells. Afterward, primary antibodies conjugated to APC, PE, AlexaFluor700, APC-AlexaFluor700, APC-eFluor780, FITC, Brilliant Violet 421, Brilliant Violet 610, Brilliant Violet 650, PerCPCy5.5, PE.Cy7, PE.CF594, or biotin were added for 25 min at 4 °C in PBS with 1% BSA (Roth, Karlsruhe, Germany), 2mM EDTA (Applichem, Vilassar de Mar, Spain), and 2mM Sodiumazide (Roth). Unless otherwise indicated, antibodies were purchased from BioLegend, San Diego, USA. The antibodies were directed against B220 (clone: RA3-6B2), c-Kit (clone: 2B8, eBio), CD4 (clone: GK1.5), CD8 (clone: 53-6.7), CD11b (clone: M1/70), CD11c (clone: N418, eBio), CD19 (clone: 6D5), CD21 (clone: 7E9), CD23 (clone: B3B4, eBio), CD38 (clone: 90, eBio), CD62L (clone: MEL-14, eBio), CD86 (clone: GL1, eBio), CD138 (clone: 281-2), Fas (clone: SA367H8), F4/80 (clone: BM8), GL7 (clone: GL7, BD), GR1 (clone: RB6-8C5), IgA (polyclonal, Southern Biotechnology, Birmingham, USA), IgD (clone: 11-26c.2a), IgM (clone: R6-60.2, BD), IgM (clone: Il/41, eBio), IgG1 (clone: A85.1, BD), Ly6G (clone: 1A8, BD), MHC II (clone: AF6-120-1), TACI (clone: ebioF10-3, eBio), and T-Bet (clone: 4B10). To identify NP-specific plasma cells, we used NP-PE (Biosearch Technologies). To detect 9-*O*-acetylation (ICV-HE^0^-biotin) and 7,9-*O*-acetylation (BCoV-HE^0^-biotin) virolectins coupled to biotin were used, which were kindly provided by Martina Mühlenhoff (Medical School of Hannover). In both cases, the esterase activity was inactivated by an alanine exchange of the active site serine (HE^0^) (3). The biotin-conjugated antibodies and the biotin-labeled virolectins were detected using streptavidin coupled to PE-Dazzle (eBio). For intracellular staining of immunoglobulins, the cells were then permeabilized with the Cytofix/Cytoperm kit (BD Biosciences, Franklin Lakes, USA). For the transcription factor T-Bet, the Foxp3/Transcription Factor Staining Buffer Set (eBio) was used according to the manufacturer’s protocol. Data were aquired with the CytoFLEX S (Beckman) and analyzed using FlowJo™ software (10.7.0). Cell sorting was performed at the cell sorting core facility of Friedrich-Alexander University, Erlangen-Nürnberg, with the MoFlo Astrios EQ 1 (Beckman Coulter, Brea, USA). The following markers were used to identify B cells (B220^+^), germinal center B cells (GL7^+^, Fas^+^), and plasma cells (CD138^+^, TACI^+^). For T cells, CD3 and the corresponding CD4 or CD8 marker were used.

### 2.4 Recombinant Siglec-E-F_c_ staining

For recombinant Siglec-E-F_c_ staining, cell preparation was carried out according to the protocol described above. After staining the cells with fixable viability dye (eBioscience) and F_c_ Block (2.4G2, hybridoma generated in-house), the cells were incubated with recombinant Siglec-E-F_c_ (1:100, BioLegend) for 30 min at 4 °C. After a washing step, the human F_c_ part was detected with an anti-human-IgG-F_c_-PE (Jackson ImmunoResearch, Cambridgeshire, UK) antibody (25 min at 4 °C). The remaining unbound F_c_ parts were blocked for 15 min at 4 °C with an unlabeled IgG rat anti-mouse IgE antibody (clone: R35-92, BD). Finally, standard surface staining was conducted, and the samples were measured on the CytoFLEX S (Beckman Coulter).

### 2.5 Naïve B cell isolation using magnetic-activated cell sorting

All of the following steps were performed under sterile conditions. After the sterile cell preparation, including the lysis of erythrocytes, B cell isolation was executed following the “B cell isolation MACS Kit” (Miltenyi Biotec, Bergisch Gladbach, Germany) manual. Briefly, the non-B cells were labeled with a biotin-labeled antibody mix, followed by antibody binding with magnetic anti-biotin beads. The cell separation was carried out using the MidiMACS separator (Miltenyi Biotec).

### 2.6 CellTrace Violet cell proliferation labeling

If needed, the isolated B cells were labeled with CellTrace Violet (CTV) Cell Proliferation Kit (1:1000, ThermoFisher, Waltham, USA) according to the manufacturer’s instructions. CTV dilution was measured 72 h after B cell stimulation *in vitro* and on day 4 after *in vitro* generation of germinal center B cells. Data were acquired with the CytoFLEX S (Beckman Coulter) and analyzed using FlowJo™ software (10.7.0).

### 2.7 *In vitro* B cell stimulation

After the naïve B cell isolation, 0.5 x 10^6^ B cells in 1 mL RPMI 1640 medium (Gibco) with 10% FCS (Gibco), 1.2 mM L-Glutamine (Gibco), 100 U/mL penicillin/streptomycin (Gibco), 1X non-essential amino acids (Gibco), 1 mM sodium pyruvate (Gibco), 50 µM beta-mercaptoethanol (Gibco) were seeded per well of a 24-well plate (Greiner). Stimulation with thymus-independent (10 µg/mL LPS (Sigma, St. Louis, USA), 5 µg/mL anti-IgM (Jackson ImmunoResearch)) or thymus-dependent (10 µg/mL anti-CD40 (clone: FGK4.5, Ichorbio, Wantage, UK), with co-stimulation using 10µg/mL IL-4 (BioLegend)) antigens was performed at 37 °C and 5% CO_2_ for 24, 48, or 72 h. To analyze B cell stimulation, flow cytometry staining was performed for the B cell activation markers MHC II and CD86. Data were acquired with the CytoFLEX S (Beckman Coulter) and analyzed using FlowJo™ software (10.7.0).

### 2.8 *In vitro* generation of germinal center B cells

In order to generate germinal center B cells *in vitro*, the system established by Nojima and colleagues was used (37). Further adaptations of the protocol were made according to the method described by Robinson and colleagues (38). This system is based on a 40LB feeder cell line (a kind gift from Daisuke Kitamura, Tokyo University of Science) that expresses CD40 ligand and secretes B cell-activating factor (BAFF). This feeder cell line was cultivated in DMEM, high glucose, and GlutaMAX (Gibco) supplemented with 10% FCS (PAN-Biotech, Aidenbach, Germany) and 100 U/mL penicillin/streptomycin (Gibco). In order to avoid 100% confluence during the culture the 40LB cells were inactivated using 10 µg/mL mitomycin C (SERVA Electrophoresis GmbH, Heidelberg, Germany) for 1.5–2 h at 37 °C one day before seeding the naïve B cells. After inactivation, 6 x 10^5^ 40LB cells per well of a 6-well plate (Greiner, Kremsmünster, Austria) were seeded in 3 mL medium. Subsequently, we followed the protocol of Meyer et al., which was established in our laboratory (39). The following day (day 0), naïve splenic B cells were MACS isolated as described above, and 8 x 10^4^ B cells per well were seeded onto the inactivated 40LB feeder cells in 6.5 mL RPMI 1640 medium (Gibco) supplemented with 10% FCS (Gibco), 10 mM HEPES (Gibco), 1 mM sodium pyruvate (Gibco), 100 U/mL penicillin/streptomycin, 50 µM beta-mercaptoethanol (Gibco), and 1 ng/mL recombinant mouse IL-4 (BioLegend). To culture the induced germinal center B cells until day 4 or longer, 4.5 mL of medium was removed and replaced with 4.5 mL of fresh RPMI 1640 medium with additives on day 3 of the culture. Cells were analyzed on days 3 and 4. To harvest the cells, 2 mM EDTA (pH 8) (Gibco) was added to the cultures, and the 6-well plates were incubated for 5 min at room temperature. Afterward, the cells were collected, stained, and analyzed using flow cytometry. Data were acquired with the CytoFLEX S (Beckman Coulter) and analyzed using FlowJo™ software (10.7.0).

### 2.9 ELISA

In order to determine the blood serum titers of NP-specific IgM and IgG1, blood was collected on day 0, 7, 14, 21, and 28 (NP-KLH) and on days 0, 5, 7, 10, and 14 (NP-Ficoll). Flat-bottom MaxiSorp 96-well plates (Greiner) were coated with 10 µg/mL NP (17)-ELISA (Biosearch Technologies) diluted in PBS (Gibco) and incubated overnight at 4 °C. To block the plates, 1% BSA (Roth) in PBS was used the next day for 2 h at 37 °C. After this, the plates were washed three times with PBS containing 0.1% Tween20 (Roth). Serum samples were pre-diluted 1:20 and then serially diluted 1:3 in PBS containing 0.1% BSA. As a standard, the sera of all animals from the last day (either day 28 or day 14) were pooled and pre-diluted 1:15. Serum samples were incubated overnight at 4 °C on the pre-coated plates. The next day, plates were washed three times with PBS containing 0.1% Tween20, followed by a 1-h incubation at 37 °C with either IgM-AP (1:5000; Southern Biotech) or IgG1-AP (1:5000; Southern Biotech). Finally, the substrate for alkaline phosphatase (4-nitrophenyl phosphate disodium salt hexahydrate tablet; Sigma), dissolved in 20 mL of a 9.7% solution of 2,2-iminodiethanol (Roth) in H_2_O and 0.5mM MgCl_2_ (pH 9.8) (Roth), was added to detect the binding of the antibodies. The optical density was measured using an ELISA reader (Tecan, Männedorf, Switzerland) at 405 nm, and the data were analyzed using SoftMax Pro (Molecular Devices, San Jose, USA).

### 2.10 ELISpot

To determine the number of NP-specific antibody-secreting cells in spleen and bone marrow of NP-KLH (day 28) and NP-Ficoll (day 14) immunized St8Sia6^-/-^ and St8Sia6^+/+^ control mice, ELISpot following the protocol of Brunner et al. (40) was performed. Therefore, flat-bottom MaxiSorp 96-well plates (Greiner) were coated overnight at 4 °C with coating buffer (15 mM Na_2_CO_3_, 35 mM NaHCO_3_ in H_2_O, pH = 9.6) containing 5 µg/mL NP(13)-BSA. The next day, a starting concentration of 2 x 10^6^ cells in 150 µL RPMI medium supplemented with 10% FCS was seeded in the first row of the plates and serially diluted 1:3. The cells were incubated overnight (37 °C, 5% CO_2_). Secondary antibodies, namely, goat anti-mouse IgM-AP (1:3000; Southern Biotech) or goat anti-mouse IgG1-AP (1:3000; Southern Biotech) in PBS with gelatin and Tween20, were added and incubated for 1 h at 37 °C. After that, the plates were again washed three times with PBS containing 0.05% Tween20, followed by addition of the ESA substrate (0.15 M AMP, 0.1 M MgCl_2_, 0.01%Triton X-405, 1% NaN_3_, 1 mg/ml BCIP). ESA substrate incubation was performed in a dark chamber at room temperature for 2–3 h until blue spots were visible. To stop the reaction, plates were washed five times with Milli-Q water (Sigma Aldrich). Plates were analyzed using the ImmunoSpotR Series 6 Ultra-V Analyzer (C.T.L., Rutesheim, Germany) together with the BioSpotR ImmunoSpot 5.1.36 (C.T.L.) software.

### 2.11 Calcium mobilization assay

To measure calcium mobilization, we followed the protocol established in our laboratory (39). A total of 1 x 10^7^ splenic cells was resuspended in 700 µL RPMI 1640 medium (Gibco) containing 5% FCS (PAN) and loaded with 0.7 µg Indo-1 acetoxymethyl ester in DMSO (Invitrogen) with 7.4% pluronic F-127 (Invitrogen, Waltham, USA) by shaking for 25 min at 30 °C protected from light. Then, 700 µL RPMI 1640 medium containing 10% FCS was added, and the whole mixture was shaken and incubated for 10 min at 37 °C. The cells were washed once and then stained with Mac-1-FITC (clone: M1/70, ebio) and CD5-PE (clone: 53-7.3, BD) as described above, or alternatively with a staining mix containing B220-PE (clone: RA3-6B2, BioLegend) and CD24-FITC (clone: M1/69, BD). After staining, the cells were washed twice and resuspended in cold Krebs-Ringer solution (DELTAMEDICA, Reutlingen, Germany). Calcium (Ca^2+^) mobilization was measured with an LSR II (BD Biosciences). First, basal Ca2^+^ levels were recorded for 50 s. Then, BCR stimulation was performed using F_(ab)2_fragment anti-IgM (Jackson ImmunoResearch), and Ca2^+^ mobilization was measured for up to 3 min. To determine loading efficiency, 5 µg ionomycin (Sigma) was added to induce maximum Ca2^+^ influx into the cells. The data were analyzed using FlowJo software (10.7.0).

### 2.12 Anti-nuclear antibodies

Serum samples were diluted 1:250 in PBS and then loaded onto a Kallestad Hep-2 Cell Line 12-well slide (BioRad, Berkely, USA). After a 30-min incubation period at room temperature, the slides were washed twice with PBS. To detect anti-nuclear antibodies, goat anti-mouse IgG-AF488 (1:500; Jackson ImmunoResearch) or goat anti-mouse IgM-AF488 (1:500; Jackson ImmunoResearch) was added for 30 min at room temperature. Lastly, the slides were embedded with DePex (OmniLab, Bremen, Germany). Measurements of the slides were conducted using the Scope.A 1 (Zeiss, Oberkochen, Germany) fluorescent microscope in combination with Axio VionRel software. Analysis was performed using ImageJ.

### 2.13 dsDNA ELISA

Flat-bottom MaxiSorp 96-well plates (Greiner) were prepared with 10 µg/mL poly-L-lysine (Sigma) in 10 mM Tris (Roth)-1 mM EDTA (Applichem, Darmstadt, Germany) buffer for 2 h at room temperature. After a washing step, plates were coated with 20 µg/mL calf thymus DNA (Sigma) overnight at 4 °C. The next day, plates were blocked with PBS containing 0.05% Tween20 and 2% FCS. Without washing, the blocking reagent was removed, and serum samples and a standard (MRL/lpr mouse serum) were added at a dilution of 1:100. The standard was further serially diluted 1:2 and incubated for 1 h at room temperature. Next, anti-mouse IgG-HRP (1:5000; Jackson ImmunoResearch) was added for 1 h at room temperature. Finally, o-phenylendiamin-dihydrochloride (Sigma) in 0.05 M disodium hydrogen phosphate (Roth), 0.05 M citric acid (Sigma), and 0.012% hydrogen peroxide (Roth) was added. Data were acquired using an ELISA reader (Tecan) at two wavelengths: 492 nm and 620 nm. Then, data were analyzed using SoftMax Pro (Molecular Devices).

### 2.14 Blood urea nitrogen test

Blood urea nitrogen testing was conducted using the BUN Reagent Set (UV-Kinetic Method) (Teco-Diagnostics, Anaheim, USA). Serum samples and an MRL/lpr serum sample as a positive control were diluted 1:150 with the enzyme reagent provided by the kit. Further, the testing was performed according to the manufacturer’s instructions. The optical density was measured using an ELISA reader (Tecan) at 600 nm, and data were analyzed using SoftMax Pro (Molecular Devices).

### 2.15 RT-PCR

Cell pellets were collected, and RNA was isolated using the RNeasy^®^ Plus Micro Kit (Quiagen, Venlon, Netherlands). After that, RNA concentration and purity were measured using NanoDrop (ThermoFisher). Then, 0.5 µg RNA per sample was used with the FastGene Scriptase II Kit (Nippon Genetics, Düren, Germany) to synthesize cDNA.

For RT-PCR, ST8Sia4 (5′-CAAGTGCGAACTGCCTAT-3′ and 5′-CTTTTCCATTCAGATCCTTGGG-3`), ST8Sia6 (5′-TCACAAACATACAGAGATGCCC-3′ and 5′-TCTTGCTTTCCACCTCGTAG-3′) and Actin (5`-CCAACTGGGACGACATGGAG-3` and 5`-CTCGTAGATGGGCACAGTGTG-3`) primers were used. As a cDNA intercalating reagent, SYBR® Green (Qiagen) was used. Data were acquired and analyzed using AriaMx Real-Time PCR (Agilent Technologies, Santa Clara, USA). To evaluate the data, ΔΔC_T_ values were determined, and the relative expression of the gene of interest to actin was calculated using 2^-ΔΔCT^.

### 2.16 Virolectin-based immunoprecipitation of *O-*acetylated sialoglycoproteins

For Western blot analysis, cells were resuspended in lysis buffer (50 mM Tris-HCl, 150 mM NaCl (Roth), 5 mM EDTA (Applichem), 2% TritonX-100 (Roth), 0.5% Sodium-deoxycholate (Sigma)) containing the following protease inhibitors: 1 mM phenylmethylsulfonylfluoride (Sigma), 1 mM sodium orthovanadate (Sigma), 5 µg/ml leupeptin (Roth), and 1 µg/ml aprotinin (Roth). Lysis was performed for 1 h on ice. Cell debris was removed by centrifugation, and the protein concentration in the cleared lysate was determined using the Pierce^®^ BCA Protein Assay Kit (Thermo Fisher). For each genotype, lysate containing 60 µg of total protein was used for virolectin-based immunoprecipitation of *O*-acetylated sialoglycoproteins. For that, Protein A Dynabeads (Thermo Fisher) were coupled with 5 µg ICV-HE^0^-hF_c_ and 5 µg BCoV-HE^0^-hF_c_ for 2 h at 4 °C in advance. After 2 h of immunoprecipitation at 4 °C, the proteins were reduced and denatured using 4x RotiLoad (Roth) for 5 min at 95 °C.

To enrich *O*-acetylated sialoglycoproteins for subsequent mass spectrometric analysis, 5.5 x 10^5^ cells per genotype were resuspended in lysis buffer containing 2 mM phenylmethylsulfonyl fluoride (Sigma) and cOmplete, EDTA-free Protease Inhibitor Cocktail (Roche, Basel, Switzerland) according to the manufacturer’s instructions. Cell debris was removed, and the lysate was pre-cleared by incubation with Protein A Dynabeads (ThermoFisher) for 1.5 h at 4 °C. The Protein A beads were removed, and the extracts were mixed with virolectin-coupled Protein A Dynabeads as described above.

### 2.17 SDS-PAGE, Coomassie-staining, and Western blot

Immunoprecipitated *O*-acetylated sialoglycoproteins were separated by 10% SDS-PAGE prior to the mass spectrometric analysis. Gels were stained overnight with Coomassie Blue (Roth) and destained by five washing steps with methanol. Each lane was cut into nine pieces for subsequent in-gel trypsin digestion as described below.

For Western blot analysis following the virolectin-based immunoprecipitation of *O*-acetylated sialoglycoproteins (Section 2.16), proteins were loaded onto a 7.5% SDS-PAGE and run as described earlier. Blotting of proteins onto a PVDF membrane (BioRad) was performed using the Trans-Blot Turbo System (BioRad). In the case of CD45, the program for high molecular weight proteins was used. The membrane was then blocked with 5% BSA in PBS containing 0.1% Tween20 and incubated with a CD45 antibody (1:1000; Cell Signaling) overnight at 4 °C. Detection was conducted with an anti-rat horseradish peroxidase (HRP) antibody (1:5000; Cell Signaling, Danvers, USA), followed by chemiluminescent development of the membrane using SuperSignal™ West Pico PLUS substrate (Thermo Fisher). For quantification of the Western blot bands, ImageJ was used.

### 2.18 Flow cytometric analysis of phosphorylated proteins

To assess B cell receptor (BCR)- and CD40-mediated signaling, naïve splenic B cells were isolated by magnetic-activated cell sorting (MACS) as described in Section 2.5. Purified B cells were incubated in RPMI medium supplemented with 50 µM β-mercaptoethanol (Gibco) for 30 min at 37 °C and subsequently stained extracellularly with anti-B220-AF700 (BioLegend) as described in Section 2.3.

Cells were then stimulated in RPMI medium containing 5% FCS (Gibco) with either 20 µg/mL anti-CD40 (IchorBio) or 20 µg/mL anti-IgM (Jackson ImmunoResearch) for 3 h at 37 °C. Following stimulation, cells were fixed using the FoxP3/Transcription Factor Staining Buffer Set (Invitrogen) to terminate signaling and prepare samples for intracellular phospho-protein analysis. Intracellular staining was subsequently performed using antibodies specific for phosphorylated AKT (pAKT; BD Biosciences) or phosphorylated ERK (pERK; BD Biosciences), followed by flow cytometric analysis.

### 2.19 MS analysis

Proteins were enriched with virolectins by immunoprecipitation and subsequently separated by SDS-PAGE as described above. Gels were stained with Coomassie Blue (Roth), and lanes were sliced into nine fractions, which were subjected to in-gel digestion with Trypsin Gold (Promega, Madison, USA) according to the manufacturer’s instructions. Extracted peptides were analyzed using an Orbitrap Fusion Lumos Tribrid Mass Spectrometer (Thermo Fisher Scientific) coupled to a nanoLC system. Peptide separation was performed by reversed-phase chromatography using acetonitrile as the organic solvent. The mass spectrometer was operated in positive ion mode, and data were acquired in data-dependent acquisition (DDA) mode with a 3-s cycle time. Full MS scans were acquired in the Orbitrap at a resolution of 120,000 over an m/z range of 350–1900, with an AGC target of 4 × 10^5^ and a maximum injection time of 50 ms. MS/MS spectra were generated by higher-energy collisional dissociation (HCD) at a normalized collision energy of 35%. Precursors with charge states of 2–7 were isolated using a 1.6 m/z window, and fragment ions were analyzed in the ion trap (rapid scan rate) with an AGC target of 1 × 10^4^ and a maximum injection time of 35 ms. Dynamic exclusion was enabled (60 s, ± 5 ppm). Precursor selection required a minimum intensity of 5,000, and isotopic peaks were excluded from fragmentation.

### 2.20 Statistical analysis

Statistical analyses were performed using GraphPad Prism software. Depending on whether the data were normally distributed according to the Shapiro–Wilk test, either the Mann–Whitney *U* test or an unpaired t-test was used to evaluate significance. Statistical comparisons are shown only where relevant. For time course analyses, a two-way ANOVA was performed. Statistical data are presented as the mean ± SD.

## 3 Results

### 3.1 Establishment and confirmation of a *St8sia6* knockout mouse model

**Figure 1:**
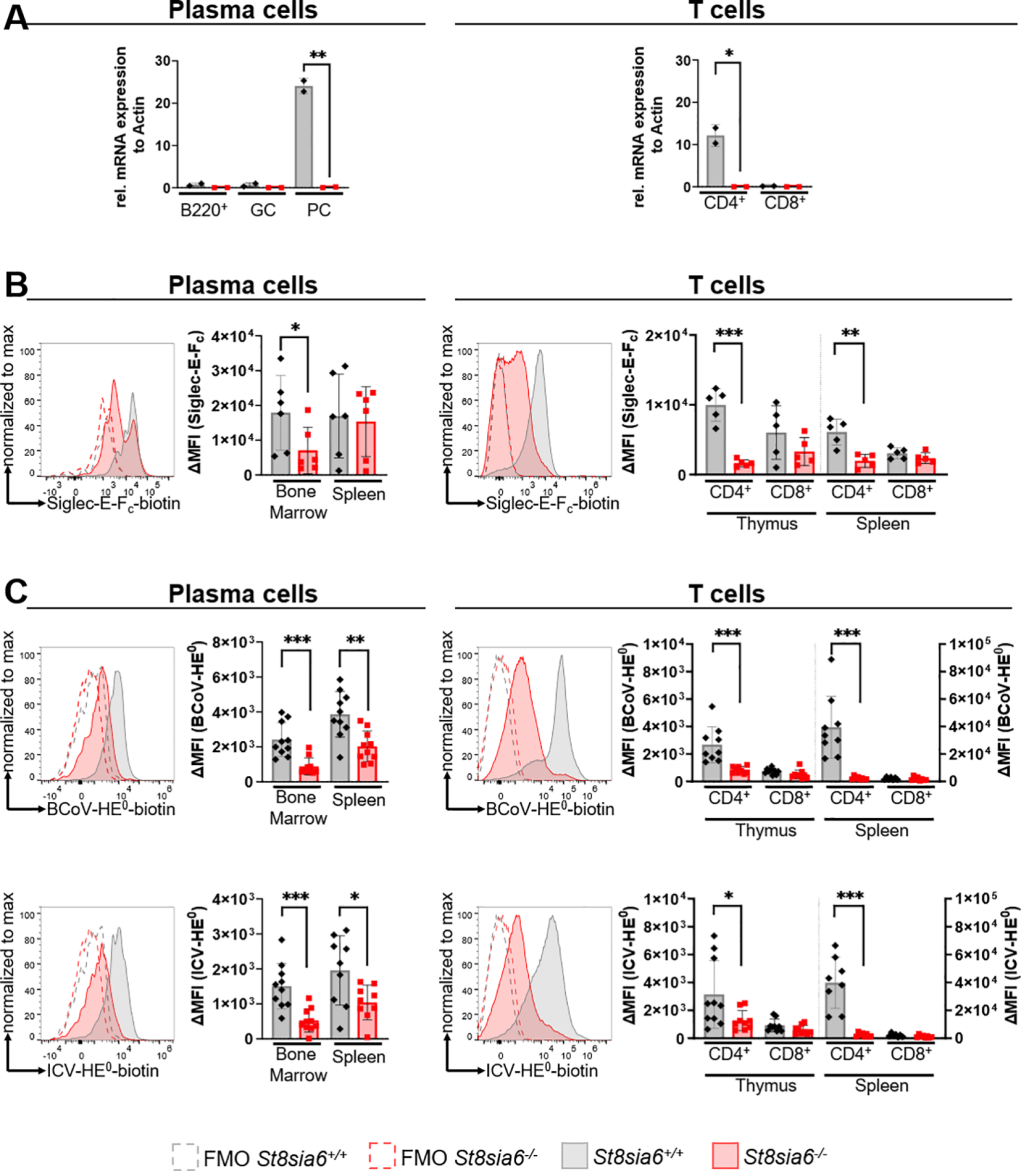
Loss of *St8sia6* in plasma cells and CD4^+^ T cells reduces Siglec-E-F_c_ binding and the display of 9-O- and 7,9-O-acetylated sialoglycans*.* **(A)** *St8sia6* RT-PCR of fluorescence-activated cell-sorted B cells (B220^+^), germinal center (GC) B cells, plasma cells (PC) (left panel) and CD4^+^ and CD8^+^ T cells (right panel) of sheep-red blood cell immunized control (gray) and *St8sia6*^-/-^ (red) mice. ΔΔC_T_ values were calculated, and the relative *St8sia6* mRNA expression to actin was determined using 2^-ΔΔCT^. **(B)** Flow cytometric analysis of recombinant Siglec-E-F_c_ binding to *St8sia6*^+/+^ (gray) and St8sia6^-/-^ (red) is depicted. Plasma cells isolated from the bone marrow and spleen (left panel), along with thymic and splenic CD4^+^ and CD8^+^ T cells (right panel), are shown. **(C)** Plasma cell analysis (left panel) depicts the binding of BCoV-HE^0^-biotin to 7,9-O-acetylation and of ICV-HE^0^-biotin binding to 9-O-acetylation in spleen and bone marrow. The biotin was detected using a streptavidin-PE Dazzle conjugate. The same staining is shown for *St8sia6*^+/+^ control and *St8sia6*^-/-^ CD4+ T cells isolated from spleen and bone marrow (right panel). In **(A–C)**, data are presented in bar charts and as means. Each dot represents one mouse. The data were collected from 2–8 independent experiments. *p < 0.05, **p < 0.01, ***p < 0.001 (Mann–Whitney test). BCoV-HE^0^, bovine Coronavirus virolectin; FMO, Full minus one control; ICV-HE^0^, influenza C virolectin; MFI, Mean Fluorescence Intensity; *St8sia6*^+/+^, wild-type control cohort.

To study the role of α-2,8-linked di-sialic acid motifs synthesized by the sialyltransferase ST8Sia6 *in vivo*, we generated a knockout mouse model by deleting exon 5 of *St8sia6* using CRISPR/Cas9. The chosen knockout strategy results in a frameshift and a premature termination codon in the mature mRNA, predicted to truncate the encoded protein (**Supplementary Figure 1**). Wang et al. demonstrated that *St8sia6* transcripts are highly upregulated in plasma cells after B cell stimulation in a T cell-independent or T cell-dependent manner (13). To validate our *St8sia6* knockout mouse model, we analyzed *St8sia6* mRNA expression levels in B cells (B220^+^), germinal center (GC) cells, and plasma cells (PC) of *St8sia6*^+/+^ control and the newly generated *St8sia6*^-/-^ mice. Compared to B220^+^ and GC cells, we observed *St8sia6* upregulation in plasma cells (PCs) of wild-type mice, but not in *St8sia6^-/-^* mice (**Figure 1A**). After isolation of CD4^+^ and CD8^+^ T cells, we found that *St8sia6* is present in wild-type CD4^+^ T cells but is expressed at very low levels in CD8^+^ T cells. The expression of *St8sia6* in CD4^+^ T cells was lost in *St8sia6*^-/-^ mice (**Figure 1A**). We further characterized our *St8sia6^-/-^* mice using recombinant Siglec-E-F_c_ staining by flow cytometry. The binding of Siglec-E-F_c_ was strongly reduced in CD4^+^ T cells in the thymus and spleen, along with in plasma cells in the bone marrow of *St8sia6^-/-^* mice (**Figure 1B**). In contrast, no reduction in recombinant Siglec-E-F_c_ staining was observed in *St8sia6*-deficient splenic plasma cells. Siglec-E can also bind to α-2,3-linked sialic acids, α-2,8-linked-diSia on gangliosides generated by ST8Sia1, and α-2,8-linked poly-sialic acids (polySia) (41–43). This may explain why not all Siglec-E-F_c_ binding was lost in our *St8sia6^-/-^* mice. To test whether *St8sia6* deficiency leads to a compensatory upregulation of ST8Sia4, the polysialyltransferase responsible for polySia biosynthesis in immune cells (44), we compared *St8sia4* mRNA expression levels in wild-type and *St8sia6^-/-^* mice. No altered mRNA expression of *St8sia4* was observed in B cells (B220^+^), GC cells, or plasma cells (PC) of *St8sia6^-/-^* mice (**Supplementary Figure 2A**).

To investigate whether ST8Sia6 drives the formation of *O*-acetylated diSia motifs, as shown previously in Chinese hamster ovary (CHO) cells (36), lymphocytes were stained with biotinylated ICV-HE^0^ and BCoV-HE^0^ for the detection of 9-*O*-acetylated and 7,9-*O*-acetylated sialoglycans, respectively. Our *St8sia6* knockout mouse model showed a strong reduction of 7,9- and 9-*O*-acetylation on CD4^+^ T cells and plasma cells (**Figure 1C**). Additionally, BCoV-HE^0^ staining of *St8sia6^+/+^* B cells revealed an increase in *O*-acetylated sialoglycans during maturation to the plasma cell stage (**Supplementary Figure 2B**). Thus, the molecular analysis of our *St8sia6^-/-^* mouse model demonstrated absence of full-length *St8sia6* transcripts (**Figure 1A**), reduced Siglec-E binding (**Figure 1B**), and a significant loss of 9- and 7,9-*O*-acetylation in immune cell populations that show *St8sia6* expression in wild-type mice (**Figure 1C**). Together, these experiments confirm a functional knockout despite the absence of a suitable antibody to demonstrate loss of ST8Sia6 protein expression.

### 3.2 *St8sia6* knockout mice show normal B cell development

**Figure 2:**
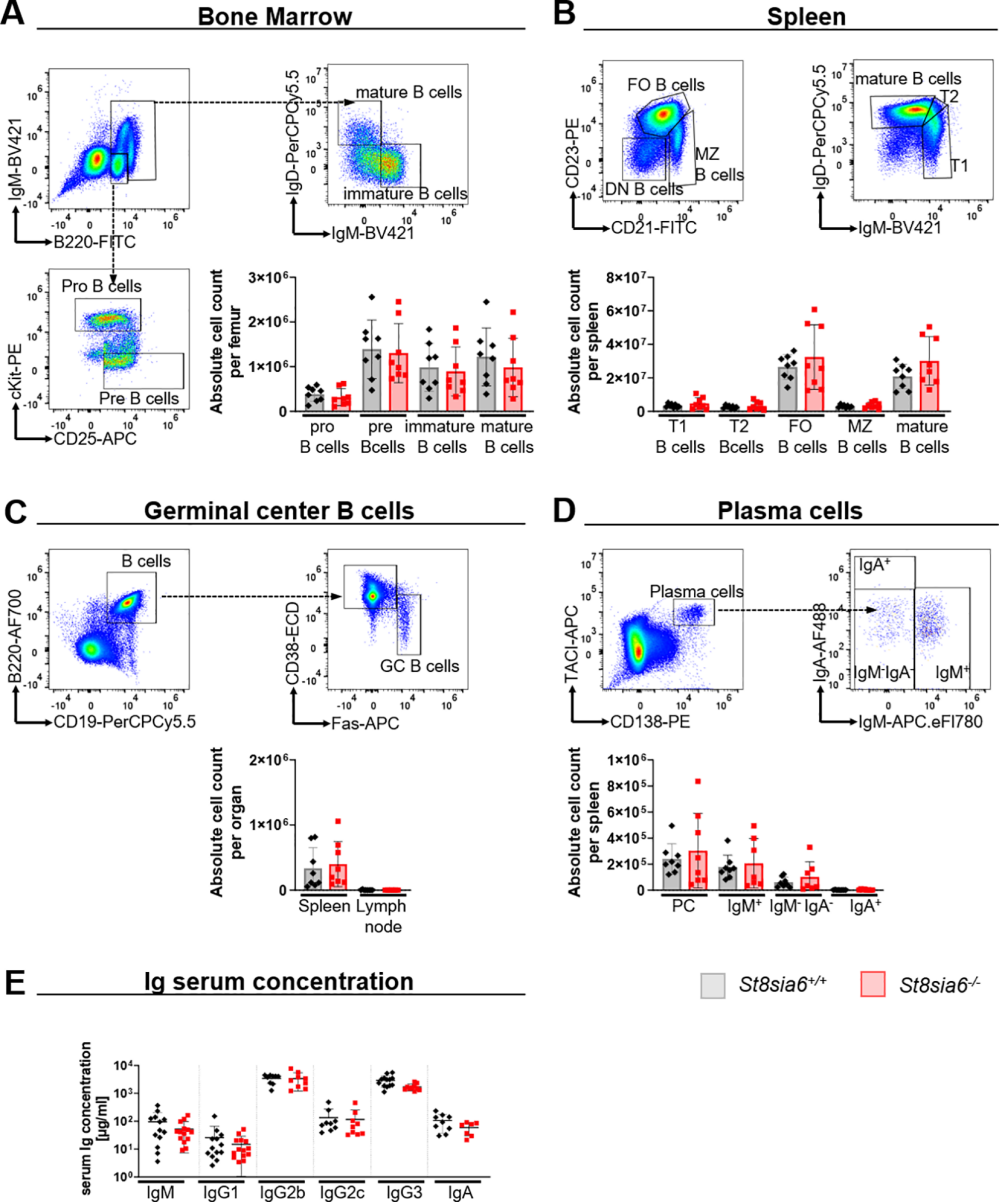
St8sia6^-/-^ mice show normal B cell development. In **(A–D),** cells from *St8sia6*^+/+^ control (gray) and *St8sia6*^-/-^ (red) mice were analyzed using flow cytometry. **(A)** Analysis of pro (IgM^-^, B220^int^, c-kit^+^), pre (IgM^-^, B220^int^, CD25^+^), immature (IgM^+^, B220^+^, IgD^-^), and mature (IgM^+^, B220^+^, IgD^+^) B cells isolated from bone marrow. **(B)** T1 (B220^+^, IgM^+^), T2 (B220^+^, IgM^+^, IgD^+^), FO (B220^+^, CD23^+^), MZ (B220^+^, CD21^+^), and mature (B220^+^, IgD^+^) B cells isolated from spleen. **(C)** Spleen and lymph nodes of *St8sia6*^+/+^ control and *St8sia6*^-/-^ mice were analyzed for GC B cell numbers (CD19^+^, B220^+^, CD38^low^, Fas^+^). **(D)** Splenic plasma cell (CD138^+^, TACI^+^) numbers, including their isotypes, as detected by intracellular staining. **(E)** Blood serum-immunoglobulin isotype concentrations of *St8sia6*^+/+^ control (black) and *St8sia6*^-/-^ (red) mice measured using ELISA. Each dot represents one mouse. In **(A–D),** data are depicted in bar charts representing the mean. The data were collected from three individual experiments including eight mice per genotype. FO, follicular; int, intermediate; MZ, marginal zone; PC, plasma cells; *St8sia6*^+/+^, wild-type control cohort; T1, transitional 1; T2, transitional 2.

It was previously discovered that during plasma cell differentiation, *St8sia6* is highly upregulated and thereby catalyzes the synthesis of di-sialic acid motifs on the plasma cell glycocalyx (13). We therefore aimed to investigate the role of these α-2,8-linked diSia motifs in plasma cell functions. To draw conclusions about plasma cells in the *St8sia6^-/-^* mouse, we first analyzed B cell development (**Figure 2**) and several other immune cell types (**Supplementary Figure 3**) in naïve *St8sia6^-/-^* mice. All analyzed precursor and B cell populations in the bone marrow (**Figure 2A**) and all B cell subpopulations in the spleen (**Figure 2B**) developed normally and were maintained at normal numbers compared with *St8sia6^+/+^* control mice. Additionally, GC B cell numbers in spleen and lymph nodes (**Figure 2C**) and splenic plasma cell numbers, including immunoglobulin-isotype distribution (**Figure 2D**) were comparable between *St8sia6^-/-^* and control mice. Furthermore, normal immunoglobulin isotype concentrations in the serum were detectable when comparing *St8sia6^+/+^* control mice and *St8sia6^-/-^* mice (**Figure 2E**). *St8sia6^-/-^* mice had normal numbers of T cells (**Supplementary Figures 3A, B**), macrophages (**Supplementary Figure 3C**), dendritic cells (**Supplementary Figure 3D**), and several other myeloid cell types (**Supplementary Figure 3E**).

### 3.3 Elevated T cell-dependent immune responses in *St8sia6* knockout mice

**Figure 3:**
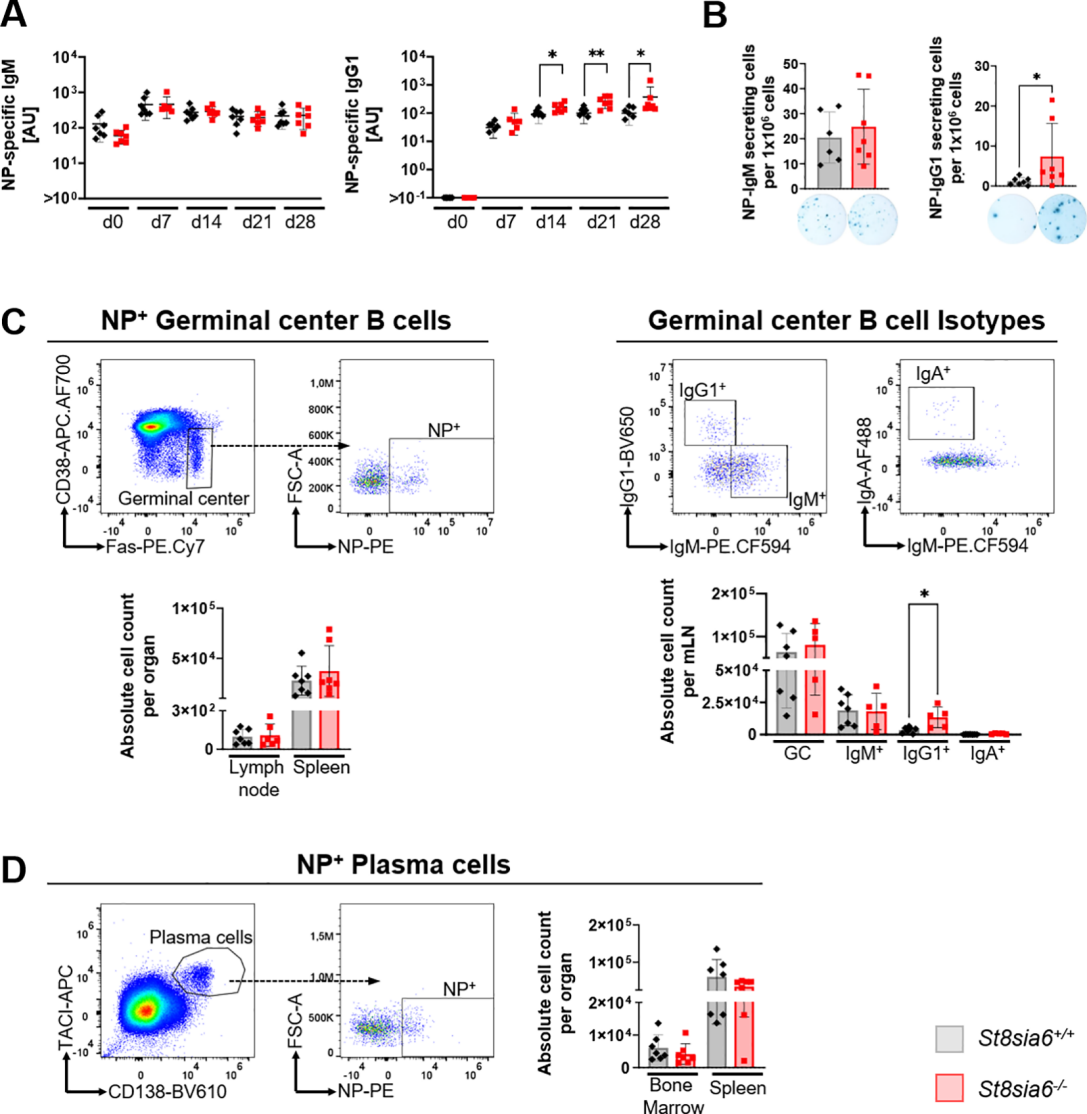
*St8sia6^-/-^* mice show increased NP-specific IgG1^+^ antibody response upon NP-KLH immunization. 28 days after i.p. immunization with 20µg NP-KLH in alum, wild-type *St8sia6*^+/+^ (gray) and *St8sia6*^-/-^ (red) mice were analyzed. **(A)** NP-specific IgM and IgG1 ELISA of serum samples collected on days 0, 7, 14, 21, and 28 of immunized *St8sia6*^-/-^ and *St8sia6*^+/+^ control mice. **(B)** ELISpot analysis on day 28 of NP-IgM- and NP-IgG1-specific antibody-producing cells in the spleen of immunized *St8sia6*^-/-^ and *St8sia6*^+/+^ mice. **(C)** Cell numbers of NP-specific GC B cells (B220^+^, CD38^-^, Fas^+^, NP^+^) and GC isotypes on day 28 were analyzed using flow cytometry. **(D)** Antigen-specific plasma cells (CD138^+^, TACI^+^, NP^+^) of spleen and bone marrow. In **(A)**, data are presented in dots. Each dot represents one mouse. **(B–D)** show the mean of the individual mice (represented as dots) in a bar chart. The data were collected from two individual experiments including seven mice per genotype. *p<0.05, **p<0.01 (Mann–Whitney test). AU, arbitrary units; GC, germinal center; i.p., intraperitoneal; mLN, mesenteric lymph node; *St8sia6*^+/+^, wild-type control cohort.

To study the immune response, we immunized *St8sia6^-/-^* mice and a *St8sia6^+/+^* control group with NP-KLH in Alum to induce a T cell-dependent immune response. Blood serum samples were analyzed over time, and the mice were sacrificed on day 28 after immunization to analyze cellular composition in several organs. While NP-specific IgM serum levels remained comparable between the control group and *St8sia6^-/-^* mice throughout the time course, mice lacking the sialyltransferase ST8Sia6 showed higher IgG1 responses specific to the NP-antigen from day 14 onward, as measured by ELISA (**Figure 3A**). ELISpot analysis on day 28 showed significantly more NP-specific IgG1 antibody-secreting cells in the spleen of *St8sia6^-/-^* mice, while the number of NP-IgM-secreting plasma cells was unchanged between the two groups (**Figure 3B**). Further analysis of NP-specific germinal center responses in lymph nodes and spleen showed no significant difference in the general number of NP-specific GC B cells (**Figure 3C**, left panel). However, germinal centers established in the mesenteric lymph nodes showed an increase in IgG1-positive GC B cells on day 28 post-immunization in *St8sia6^-/-^* mice compared with *St8sia6^+/+^* control mice (**Figure 3C**, right panel). Determination of the absolute number of NP-specific plasma cells in spleen and bone marrow showed equal numbers in both genotypes (**Figure 3D**). Of note, this NP-surface staining detects IgM^+^ and IgA^+^ plasma cells, but not IgG-secreting plasma cells, as IgG is usually not expressed on the cell surface (45–47). In conclusion, we observed more NP-specific IgG1-secreting plasma cells in the spleen, correlating with higher levels of NP-specific IgG1 in the serum and demonstrating an elevated immune response in *St8sia6^-/-^* mice compared with wild-type *St8sia6^+/+^* control mice.

To investigate immune responses further and determine whether *St8sia6^-/-^* mice exhibit B-cell intrinsic alterations in immune responses, we performed T cell-independent immunization using NP-Ficoll. *St8sia6^-/-^* mice showed normal NP-specific IgM and IgG3 responses, along with normal numbers of NP-specific IgM and IgG3 antibody-secreting cells on day 14 after immunization with NP-Ficoll. (**Supplementary Figure 4**). Thus, with this type of antigen, no differences compared to *St8sia6^+/+^* control mice could be detected.

### 3.4 *St8sia6^-/-^* B cells do not show a higher activation, but a stronger proliferation when stimulated under T cell-dependent conditions *in vitro*

**Figure 4:**
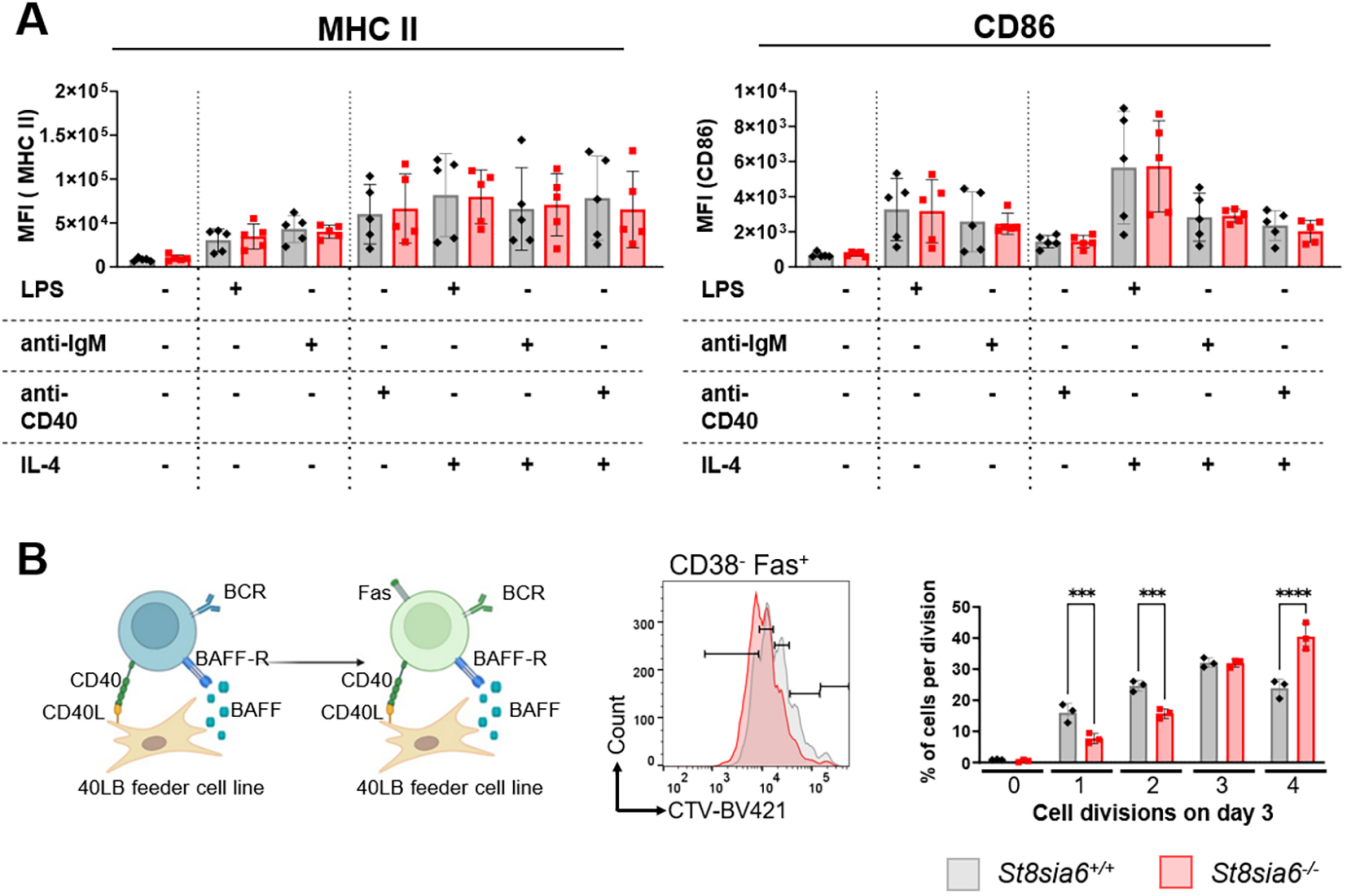
*St8sia6^-/-^* B cells show increased proliferation upon T cell-dependent stimulation. **(A)** Analysis of B cell activation markers MHC-II and CD86–48 h after *in vitro* stimulation of *St8sia6*^+/+^ control (gray) and *St8sia6*^-/-^ (red) B cells with LPS, anti-IgM, and anti-CD40 with or without costimulation with IL-4. **(B)** Proliferation on day 4 of induced germinal center culture *in vitro* of *St8sia6*^+/+^ (gray) and *St8sia6*^-/-^ (red) B cells (37). Schematic overview of the culture system (left panel), representative overlay of induced germinal center B cell proliferation of *St8sia6*^+/+^ control and *St8sia6*^-/-^ mice on day 3 in the culture (middle panel). On the right panel, the percentage of cells per cell division is indicated on the y-axis, while the number of divisions reached on day 3 is displayed on the x-axis. **(A, B)** show the data in bar charts. Each dot represents one mouse. **(A)** shows data from three individual experiments with five mice per genotype, **(B)** the data from two individual experiments with three mice. ***p<0,001, ****p<0,0001 (2-way ANOVA). CTV, CellTrace Violet®; MFI, Mean Fluorescence Intensity; *St8sia6*^+/+^, wild-type control cohort; TD, T cell dependent; TI, T cell independent.

In order to study B cell signaling and activation in *St8sia6^-/-^* mice, we first analyzed anti-BCR-induced B cell signaling. When stimulated with two different concentrations of anti-IgM F_(ab)2_-fragments, *St8sia6^-/-^* and control B cells were able to mobilize equal amounts of calcium after B cell receptor stimulation (**Supplementary Figure 5A**). To study B cell activation *in vitro*, isolated splenic B cells were stimulated with various stimuli, and MHC-II or CD86 upregulation was analyzed. *St8sia6^-/-^* and *St8sia6^+/+^* control B cells showed similar upregulation of these two activation markers under all examined stimulation conditions (**Figure 4A**). We also determined B cell proliferation after stimulation *in vitro* using CellTrace Violet (CTV) dilution assays. After 72 h, *St8sia6^-/-^* B cells had divided more after anti-IgM plus anti-CD40 stimulation, as indicated by lower mean fluorescence intensity (MFI) of CTV and a higher percentage of cells with four divisions (**Supplementary Figure 5B**). Anti-IgM or LPS stimulation, however, did not induce stronger proliferation of *St8sia6^-/-^* B cells (**Supplementary Figure 5C**). In order to exclude changes in CD40 signaling, we analyzed downstream phosphorylation of pAKT and pERK upon anti-CD40 or anti-IgM stimulation (**Supplementary Figure 5D**). This analysis revealed no differences, which demonstrates unaltered intracellular signaling.

In order to examine a later activation state of B cells, we used the induced germinal center system (37), in which B cells differentiate to GC-like cells *in vitro* by co-culture with a CD40L-expressing and BAFF-secreting feeder cell line (**Figure 4B**). B cells were labeled with CTV before seeding, and after co-culture, cell division peaks with diluted CTV were identified and quantified by flow cytometry on day 3. Within this system, we observed that *St8sia6^-/-^* GC-like B cells proliferated more than wild-type control cells, as indicated by a lower percentage of *St8sia6^-/-^* cells with one or two divisions, but a higher percentage of *St8sia6^-/-^* cells with four divisions (**Figure 4B**).

Overall, B cell activation in *St8sia6^-/-^* mice appeared unaltered with regard to activation marker expression and signaling responses after B cell receptor stimulation. Nevertheless, *St8sia6*-deficient B cells showed increased proliferation upon B cell activation or, as GC-like B cells, when stimulated under T cell-dependent conditions.

### 3.5 60-week-old *St8sia6^-/-^* mice show enlarged populations of activated B cells and elevated levels of anti-nuclear IgG autoantibodies

**Figure 5:**
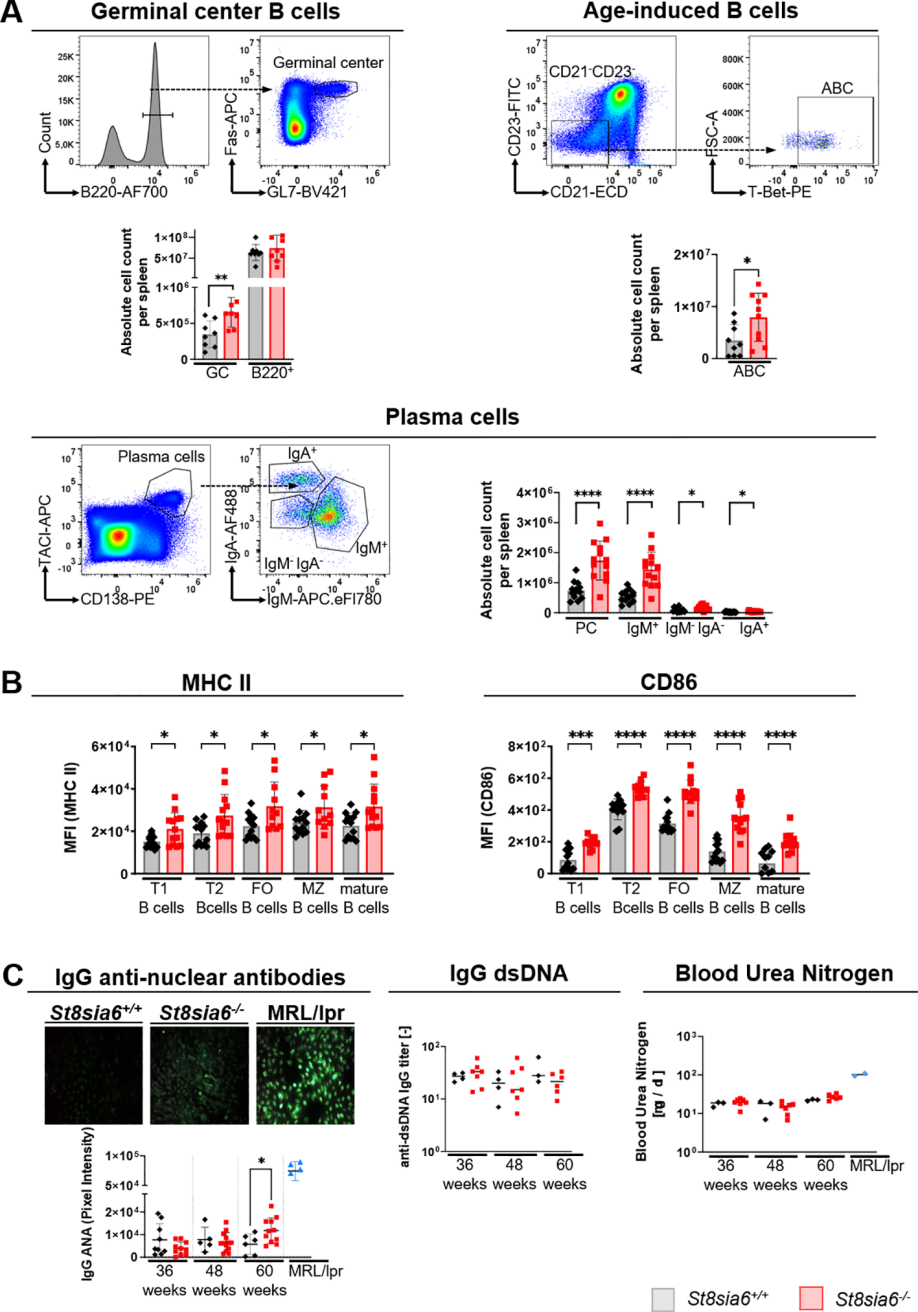
60-week-old *St8sia6^-/-^* mice show enlarged germinal center B cells, age-induced B cells, plasma cell populations, and higher B cell activation markers. **(A)** Analysis of splenic germinal center B cells (B220^+^, GL7^+^, Fas^+^), plasma cells (CD138^+^, TACI^+^), and age-induced B cells (B220^+^, CD21^-^, CD23^-^, T-Bet^+^) in 60-week-old *St8sia6*^-/-^ mice (red) and *St8sia6*^+/+^ (gray) control mice using flow cytometry. **(B)** Mean fluorescence intensity of the activation markers MHC II and CD86 on different splenic B cell subsets in 60-week-old *St8sia6*^-/-^ mice in comparison to *St8sia6*^+/+^ control mice. **(C)** Testing for autoimmunity by analyzing IgG anti-nuclear antibodies (ANA), anti-double-stranded DNA IgG titers, and blood urea nitrogen levels in blood serum of *St8sia6*^-/-^ and *St8sia6^+/+^* mice. **(A–C)** depict the data in bar charts reflecting the mean between the different mice (represented as dots). The graph shows three individual experiments with 7–11 mice per genotype. *p<0.05, **p<0,01, ***p<0,001, ****p<0,0001 (Mann–Whitney test and unpaired t-test). GC, germinal center B cells; MFI, Mean Fluorescence Intensity; PC, plasma cells; *St8sia6*^+/+^, wild-type control cohort.

It was previously shown that overexpression of *St8sia6* promotes immune tolerance during inflammation and autoimmunity in a mouse model of diabetes (6). Based on this, we wanted to investigate whether the loss of ST8Sia6-mediated α-2,8-linked diSia motifs can lead to spontaneous autoimmunity with age. Thus, we analyzed 60-week-old naïve *St8sia6^-/-^* and *St8sia6^+/+^* control mice. Interestingly, *St8sia6^-/-^* mice showed enlarged germinal center, plasma cell, and age-induced B cell populations (**Figure 5A**). Age-induced B cells (ABCs), which are CD23^-^ CD21^-^ double-negative and express T-Bet, are often found to accumulate in autoimmune diseases (48, 49). In general, populations of all plasma cell (PC) Ig isotypes (IgM^+^, IgA^+^, and IgM^-^ IgA^-^) were enlarged in 60-week-old *St8sia6^-/-^* mice. The most drastic enlargement was observed in the IgM^+^ plasma cell population.

While total B cell numbers, determined as B220^+^ cells, were not altered in their population size, B cells in 60-week-old *St8sia6^-/-^* mice showed elevated activation markers (MHC II and CD86). This phenotype of increased B cell activation was not restricted to one subpopulation but was observed across all splenic B cell subpopulations (**Figure 5B**).

Next, we aimed to investigate typical signs of autoimmune diseases, such as autoantibodies directed against nuclear structures (ANA) or double-stranded DNA (dsDNA), which are characteristic of systemic lupus erythematosus (SLE), in the serum of 60-week-old *St8sia6^-/-^* and *St8sia6^+/+^* control mice (50, 51). Moreover, we checked the urea nitrogen concentration in the blood of our experimental cohort, since these levels are known to be elevated in mice with lupus-like disease, for example, in MRL/lpr mice, which are prone to developing SLE (52). In 60-week-old female *St8sia6^-/-^* mice, we observed an accumulation of IgG ANA autoantibodies in the serum (**Figure 5C**, left panel). Testing for anti-dsDNA IgG antibodies and blood urea nitrogen levels showed no differences between the *St8sia6^+/+^* control cohort and the *St8sia6^-/-^* mice (**Figure 5C**, middle and right panel).

### 3.6 Identification of CD45 as a potential carrier of *O*-acetylated α-2,8-linked diSia motifs

**Figure 6:**
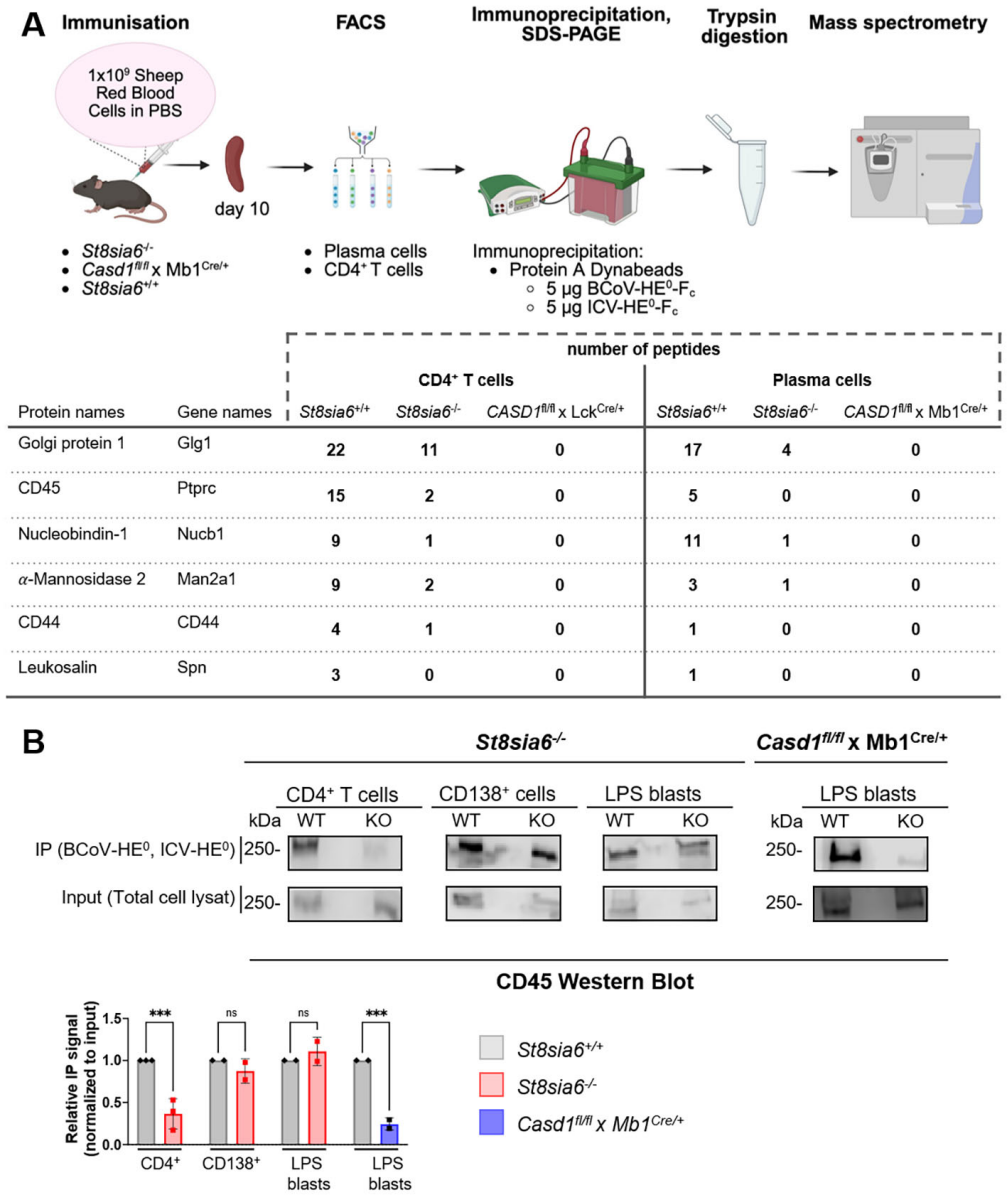
Specific enrichment revealed several glycoproteins, comprising CD45 as a potential carrier of *O*-acetylated, di-sialic acid-bearing glycans on membranes of plasma cells. **(A)** Experimental setup for carrier protein identification. *St8sia6*^+/+^ control mice, *St8sia6*^-/-^ mice, *Casd1*^fl/fl^ x Lck^Cre/+^ (lacking 9-O-acetylation on T cells), and *Casd1*^fl/fl^ x Mb1^Cre/+^ mice (lacking 9-O-acetylation on B cells) were immunized with 1 x 10^9^ sheep red blood cells i.p. After 10 days, spleens of the three different mouse strains were harvested to isolate CD4^+^ T cells and CD138^+^/TACI^+^ plasma cells using fluorescence-activated cell sorting. After immunoprecipitation with the virolectins BCoV-HE^0^-F_c_ and ICV-HE^0^-F_c_, captured proteins were separated by SDS-PAGE, stained with Coomassie, and peptides derived upon in-gel digestion with trypsin were subjected to mass spectrometry. Spectral counting suggests exclusive enrichment for several glycoproteins in mice positive for *St8sia6* and *Casd1*. **(B)** Immunoprecipitation of CD4^+^ T cells, CD138^+^ PC or LPS blasts of *St8sia6*^+/+^and *St8sia6*^-/-^ mice (left panel) or *Casd1^fl^*^/fl^ x Mb1^Cre/+^ mice (right panel), respectively, was performed with the virolectins BCoV-HE^0^-F_c_ and ICV-HE^0^-F_c_. This was followed by anti-CD45 Western blots. As a loading control, the total cell lysate before IP was also separated by SDS-PAGE and stained with anti-CD45. For each sample quantification, the intensity of the bands was analyzed using ImageJ. The intensity of the IP band was divided by the respective total cell lysate band. Because of substantial experimental variation, each wild-type ratio was set to 1 and compared to the respective knockout ratio. This graph shows representative data and quantification of 2–3 experiments. BCoV-HE^0^, bovine Corona Virolectin; ICV-HE^0^, Influenza C virolectin; KO, knockout; PC, plasma cells; WT, *St8sia6*^+/+^ wild-type control.

ST8Sia6 catalyzes the formation of α-2,8-linked diSia preferentially on *O*-linked glycoproteins (53), but the identity of diSia-carrier proteins in immune cells remains elusive. Since ST8Sia6 drives the formation of 9-*O*- and 7,9-*O*-acetylated sialoglycans in a CASD1-dependent manner (36), we performed virolectin-based enrichment of *O*-acetylated sialoglycoproteins from *St8sia6^+/+^* control, *St8sia6^-/-^*, and *Casd1*-deficient immune cells, followed by mass spectrometric analysis to identify proteins of ST8Sia6-mediated di-sialylation. CD4^+^ T cells and plasma cells were FACS-isolated from immunized *St8sia6^+/+^* control and *St8sia6*^-/-^ mice. Corresponding *Casd1*-deficient cells were isolated from immunized conditional *Casd1* knockout mice, using *Casd1*^fl/fl^ x Lck^Cre/+^ and *Casd1*^fl/fl^ x Mb1^Cre/+^ mice as T cell- and B cell-specific knockout lines, respectively. *O*-acetylated sialoglycoproteins were captured from cell extracts using the virolectins ICV- and BCoV-HE^0^ co-immobilized to magnetic beads. Virolectin-captured proteins were separated by SDS-PAGE, and each lane was excised into multiple gel pieces, which were subjected to in-gel trypsin digestion prior to LC-MS/MS-based proteomic analysis (**Figure 6A** top). This approach enabled the enrichment of several proteins from wild-type cell extracts that were either absent or detected only with reduced peptide abundance in extracts derived from *St8sia6*^-/-^ cells. Notably, none of the identified proteins in CD4^+^ T cells and plasma cells were detected in cell extracts from *O*-acetyltransferase-negative *Casd1*^-/-^ cells, thereby validating the specificity of the virolectin-based enrichment (**Figure 6A**, bottom). We identified three cell surface glycoproteins (CD45, CD44, and CD43/leukosalin) that are known to be heavily *O*-glycosylated and may thus serve as acceptors for ST8Sia6-mediated di-sialylation. The complete set of identified peptides is shown in **Supplementary Figure 6**.

We further characterized CD45 (protein tyrosine phosphatase receptor type C, PTPRC) as a potential first candidate (**Figure 6B**). After immunoprecipitation (IP) with the virolectins BCoV-HE^0^-F_c_ and ICV- HE^0^-F_c_, significantly less CD45 was pulled down from *St8sia6*^-/-^ CD4^+^ T cell extracts compared with the wild-type control, although CD45 was equally present in the lysates of CD4^+^ T cells from both genotypes (**Figure 6B**, input left panel). However, this result could not be reproduced in plasma cells (CD138^+^), where a similar level of CD45 was precipitated by the virolectins in St8Sia6^+/+^ control and St8Sia6^-/-^ plasma cells. We also generated LPS blasts for 72 h *in vitro* from St8Sia6^+/+^ control and St8Sia6^-/-^ B cells, and we did not observe differences by virolectin-precipitation of CD45 (**Figure 6B**, left panel). This finding may indicate that, in plasma cells, Sia *O*-acetylation is not restricted to α-2,8-linked diSia motifs generated by ST8Sia6 but occurs predominantly on sialylated structures formed by a distinct sialyltransferase, such as ST8Sia4. To investigate whether CD45 is also a carrier for *O*-acetylated Sia in plasma cells, we generated LPS blasts from *Casd*1^fl/fl^ x Mb1^Cre/+^ B cells lacking the *O*-acetyltransferase CASD1 and from corresponding *Casd1*^fl/fl^ control B cells. Again, we performed virolectin-based enrichment of *O*-acetylated sialoglycoproteins followed by Western blot analysis using an anti-CD45 antibody (**Figure 6B**, right panel). In this case, we observed a clear reduction in the amount of CD45 that was pulled down by virolectins from the cell extract of *Casd1* knockout LPS blasts compared with the wild-type control. The quantification of the various IPs is shown at the bottom of **Figure 6B**. Of note, CD45 expression was equal in the total cell lysates of wild-type and *Casd1* knockout LPS blasts. Taken together, these findings indicate that CD45 carries *O*-acetylated Sia in CD4^+^ T cells and LPS blasts.

## 4 Discussion

The sialyltransferase ST8Sia6, which catalyzes the synthesis of α-2,8-linked di-sialic acid motifs on immune cells, is highly expressed in CD4^+^ T cells and plasma cells, but only weakly expressed in CD8^+^ T cells and B cells, as confirmed in this study. Our *St8sia6^-/-^* mouse line showed a clear reduction of *St8sia6* mRNA expression in CD4^+^ T cells and plasma cells. The loss of cell surface expression of α-2,8-linked di-sialic acids was assessed using two methods. Since α-2,8-linked di-sialic acids create ligands for Siglec-E (6, 30), we stained CD4^+^ T cells and plasma cells with Siglec-E-F_c_. This staining revealed a loss of Siglec-E binding in bone marrow plasma cells and thymic and splenic CD4^+^ T cells of *St8sia6^-/-^* mice. Nevertheless, the reduction in splenic plasma cells was not as drastic as in CD4^+^ T cells. Siglec-E binds preferentially α-2,8-linked Sia, including α-2,8-linked diSia motifs, but also α-2,8-linked-polySia. ST8Sia4, a sialyltransferase catalyzing α-2,8-linked polySia, is also highly upregulated in plasma cells (54). Additionally, Siglec-E can bind α-2,3- and α-2,6-linked sialic acids (7, 41, 42). Therefore, the Siglec-E-F_c_ binding might not be completely lost from plasma cells of *St8sia6^-/-^* mice. Based on previous findings demonstrating that transfection-mediated expression of ST8SIA6 in CHO cells induces the formation of 7,9- and 9-*O*-acetylation on sialoglycans (36), we used two biotinylated virolectins, ICV-HE^0^ and BCoV-HE^0^, to detect this Sia modification. Virolectin staining confirmed a loss of 7,9- and 9-*O*-acetylated sialoglycans in both CD4^+^ T cells and plasma cells of *St8sia6^-/-^* mice. These findings demonstrate that the *St8sia6^-/-^* mouse line described here has lost cell surface expression of α-2,8-linked diSia motifs on the analyzed immune cells.

The B cell and plasma cell compartments in our *St8sia6^-/-^* mouse model were established normally. This was expected, since the knockout of other sialyltransferases synthesizing α-2,3-, α-2,6-, or α-2,8-linked sialic acids did not alter the development or the size of B cell populations (54–57). However, when B cells lack all sialoglycans on the surface, B cell development and survival are drastically altered, as observed in cytidine monophosphate sialic acid synthase (CMAS) conditional knockout mice (58). Therefore, the lack of single sialyltransferases may be compensated for by expression of other sialyltransferases to avoid an altered B cell development. Nevertheless, no compensatory upregulation of the related sialyltransferase ST8Sia4 could be observed on the mRNA level in our *St8sia6^-/-^* mouse model.

B cell signaling analysis showed no difference to control mice, and B cell activation marker (MHC-II and CD86) upregulation remained unchanged between *St8sia6^-/-^* and *St8sia6^+/+^* B cells. Stimuli such as LPS or anti-IgM did not induce altered proliferation, and T-cell-independent antigens elicited a completely normal antibody response. Additionally, no differences in downstream signaling pathways after stimulation with anti-IgM or anti-CD40 antibodies were observed. However, after T cell-dependent B cell stimulation *in vitro* with stimuli such as anti-CD40 or in induced GC cultures, activated *St8sia6^-/-^* B cells exhibited increased proliferation. Interestingly, this advantage in the proliferation of *St8sia6^-/-^* B cells was observed only under the involvement of the CD40/CD40 ligand (CD40L) axis. These findings suggest dysregulated immune activation possibly caused by increased T cell help, leading to increased B cell proliferation and resulting in the accumulation of activated B cells with age.

In addition, after challenging *St8sia6^-/-^* mice with T cell-dependent antigens, we observed higher antigen-specific IgG1 antibody responses and more IgG1-secreting plasma cells in our *St8sia6^-/-^* mice. Antigen-specific IgM responses were not affected; only antigen-specific IgG1 was increased, suggesting a stronger T-cell help, which is crucial for class switching. T-cell help is provided by CD40L/CD40 activation and also by IL-4 supply for B cells (59, 60). This supports our *in vitro* finding, showing higher proliferation of *St8sia6^-/-^* B cells after the introduction of T-cell-dependent stimuli. The CD4^+^ T cell population that provides help for Ig class switch in GCs is T-follicular helper cells (T_FH_ cells) (60). T_FH_ cell numbers were unchanged during TD immunization; however, their function may have been altered by the loss of α-2,8-linked di-sialic acids. This would suggest that their function is usually inhibited by these ligands. The mechanism is not known but would be independent of Siglecs, as T cells do not express any Siglecs (61). Sialic acids, as ligands for Siglecs, can regulate and dampen the immune response after activation through their ITIM domains in order to avoid overactivation (62). In the case of ST8Sia6-mediated di-sialic acids, Siglec-E has been described as a receptor (28). Siglec-E is present on monocytes, macrophages, and dendritic cells (31). Dendritic cells act as antigen-presenting cells; the lack of di-sialic acid motifs, which are also present on dendritic cells, might prevent immune regulation via the Siglec-E axis. Thus, stronger dendritic cell activation and antigen presentation due to the missing Siglec-E inhibition *in cis* could lead to stronger T_FH_ cell activation and increased class switching to IgG1. Hence, we conclude that this might not be an exclusively B cell-intrinsic phenotype but rather a result of interactions with more highly activated T cells or DCs.

In this study, 60-week-old *St8sia6^-/-^* mice exhibited a highly activated cell phenotype, with increased expression of the B cell activation markers MHC II and CD86 in all B cell subpopulations in the spleen. Moreover, the populations of GC B cells, age-associated B cells, and plasma cells were enlarged. Increased numbers of GC B cells and plasma cells are typical in mouse models in which SLE-like autoimmune disease is observed (63, 64). Age-induced B cells are often found in higher numbers in several autoimmune diseases (48, 49). Aging *St8sia6^-/-^* mice showed increased levels of ANA autoantibodies but no increase in anti-dsDNA IgG. Thus, there were clear markers of ongoing SLE-like autoimmune disease but no elevation of the typical anti-dsDNA autoantibodies (50, 51). Additionally, it was shown in a different study that overexpression of *St8sia6* rescued the spontaneous development of type 1 diabetes, which is a T cell-mediated autoimmune disease (6). Taken together with the results shown in our study, these data suggest that ST8Sia6-mediated di-sialic acid expression prevents the development of spontaneous autoimmune diseases with age.

We identified only a limited number of cell surface proteins as potential carriers of *O*-acetylated α-2,8-linked di-Sia motifs using a virolectin-based immunoprecipitation approach followed by mass spectrometry analysis. We focused on the cell surface protein CD45, which was identified in both splenic CD4^+^ T cells and plasma cells. CD45 is known to be highly glycosylated and contains many O-glycosylation sites (65). Additionally, it occurs in many different alternatively spliced isoforms. One isoform, CD45RA, is also known as B220, which is a marker restricted to B cells. B220 is the longest isoform of CD45, whereas T cells carry the shortest isoform (CD45RO) (66). CD45 is involved in inducing both BCR and TCR signaling by activating the crucial intracellular Src kinases Lyn and Lck (67, 68). An early study from the Varki lab described 9-*O*-acetylated sialomucins as markers of murine CD4^+^ T cells (69), but information on the linkage of the *O*-acetylated Sias was missing. *St8sia6* has recently been identified as one of five signature genes of the murine CD4^+^ single positive (SP) T cell lineage that is, together with other lineage-associated genes, repressed in the CD8^+^ SP lineage (70, 71). CD45 was precipitated by virolectins from CD4^+^ T cells and plasma cells of control mice but not from *Casd1*^-/-^ mice. Virolectin-based enrichment of *O*-acetylated sialoglycoproteins failed to isolate CD45 from *St8sia6*-negative CD4^+^ T cells; however, it still enriched CD45 from *St8sia6*-negative plasma cells. This finding indicates the presence of *O*-acetylated Sia residues on CD45 that have been added by a sialyltransferase distinct from ST8Sia6. This hypothesis is supported by the Siglec-E-F_c_ staining of *St8sia6^-/-^* plasma cells, in which the staining was not reduced in the spleen, while we observed a reduction in *St8sia6^-/-^* bone marrow plasma cells. Additionally, the staining of plasma cells with the BCoV- and ICV-derived virolectins showed a stronger reduction in *St8sia6^-/-^* plasma cells of the bone marrow compared to the spleen. Nevertheless, CD45 is a strong candidate for being functionally involved in inhibiting B- or T cell activation by carrying α-2,8-linked di-sialic acids, as it is a crucial cell surface protein for the initiation of lymphocyte signaling.

All in all, we conclude that α-2,8-linked di-Sia motifs synthesized by ST8Sia6 on immune cells fine-tune the control of T cell-dependent antibody responses, which is essential for preventing autoimmunity in aged mice.

## Data Availability

The original contributions presented in the study are included in the article/**Supplementary Material**. Further inquiries can be directed to the corresponding author.
