# Supplementary figures and images for: Loss of ST8Sia6 mediated α-2,8-linked di-sialylation enhances T cell-dependent antibody responses and promotes autoimmunity in aged mice
Source: Front Immunol. 2026 Jul 2;17:1874115. doi: 10.3389/fimmu.2026.1874115 (PMC13372644; doi:10.3389/fimmu.2026.1874115)

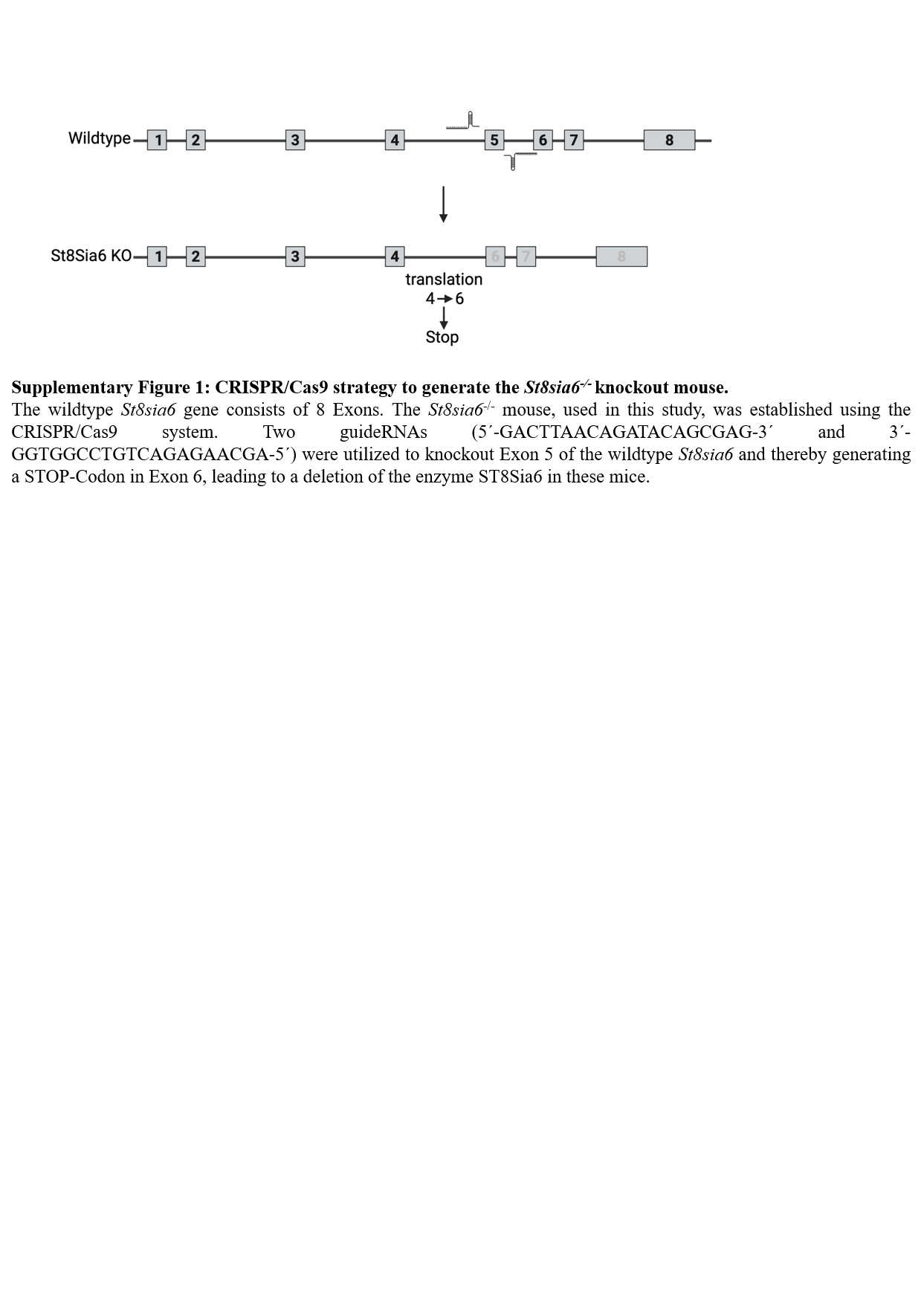

Supplement: Supplementary file 2 [file Image1.tif]

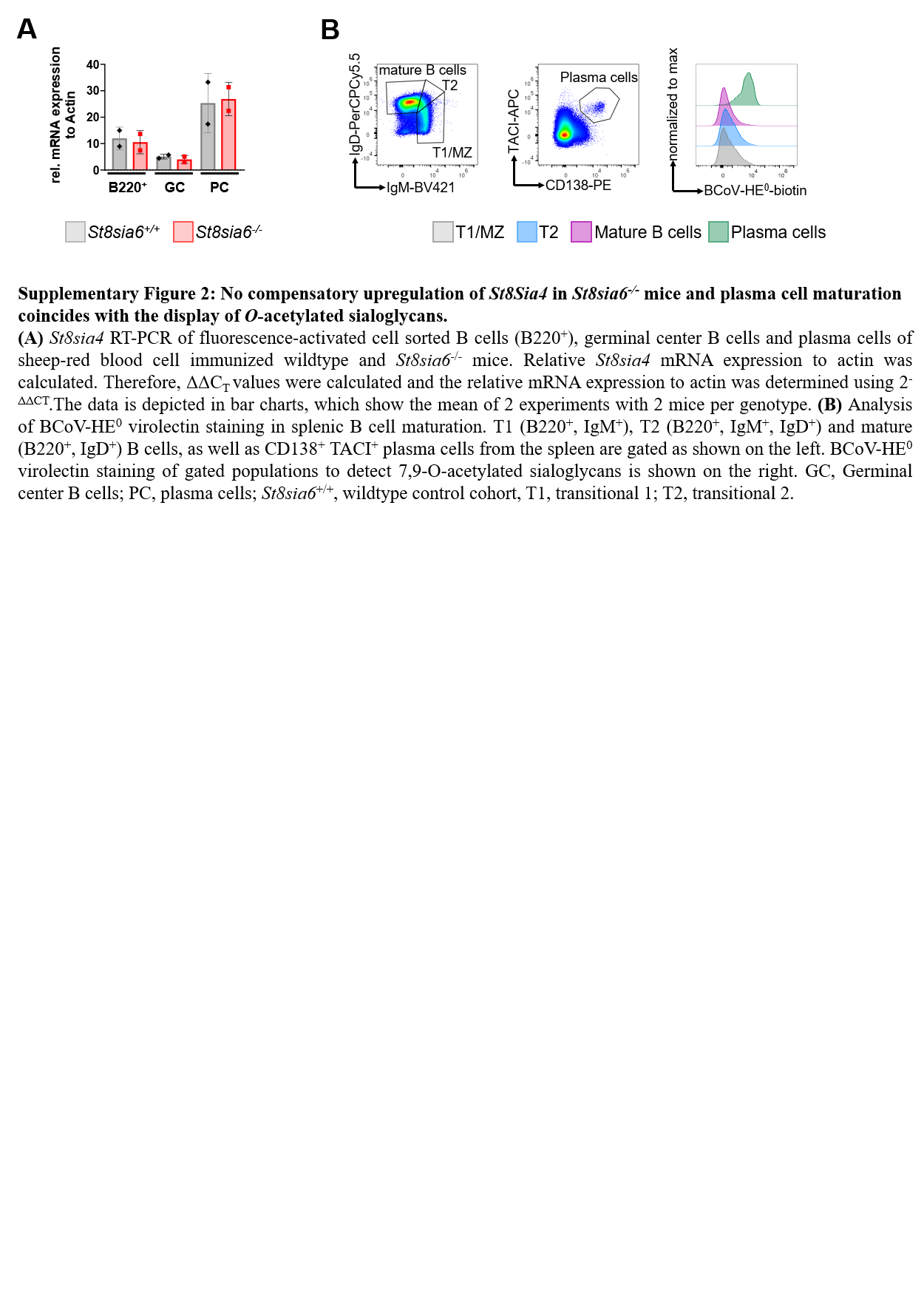

Supplement: Supplementary file 3 [file Image2.tif]

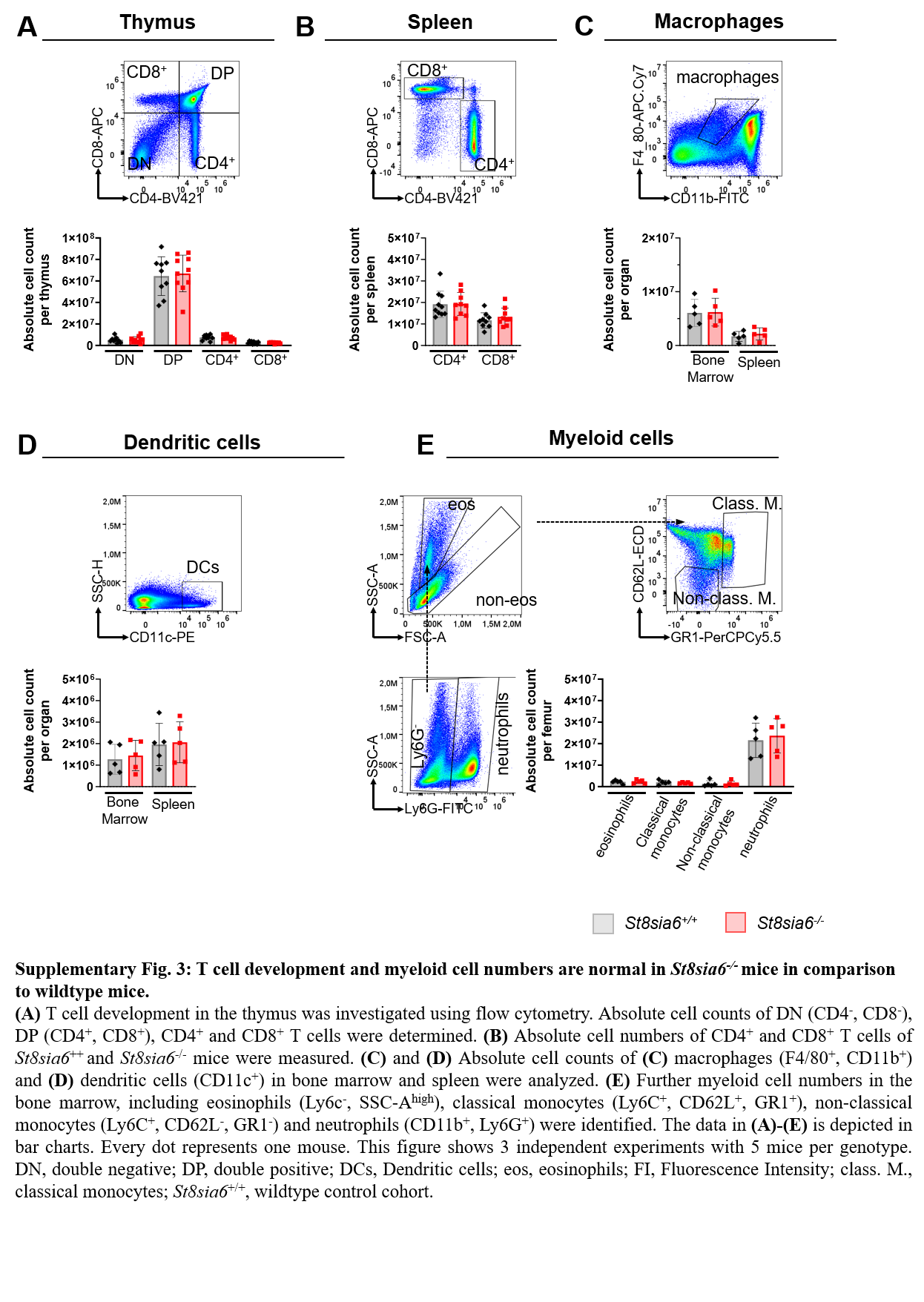

Supplement: Supplementary file 4 [file Image3.tif]

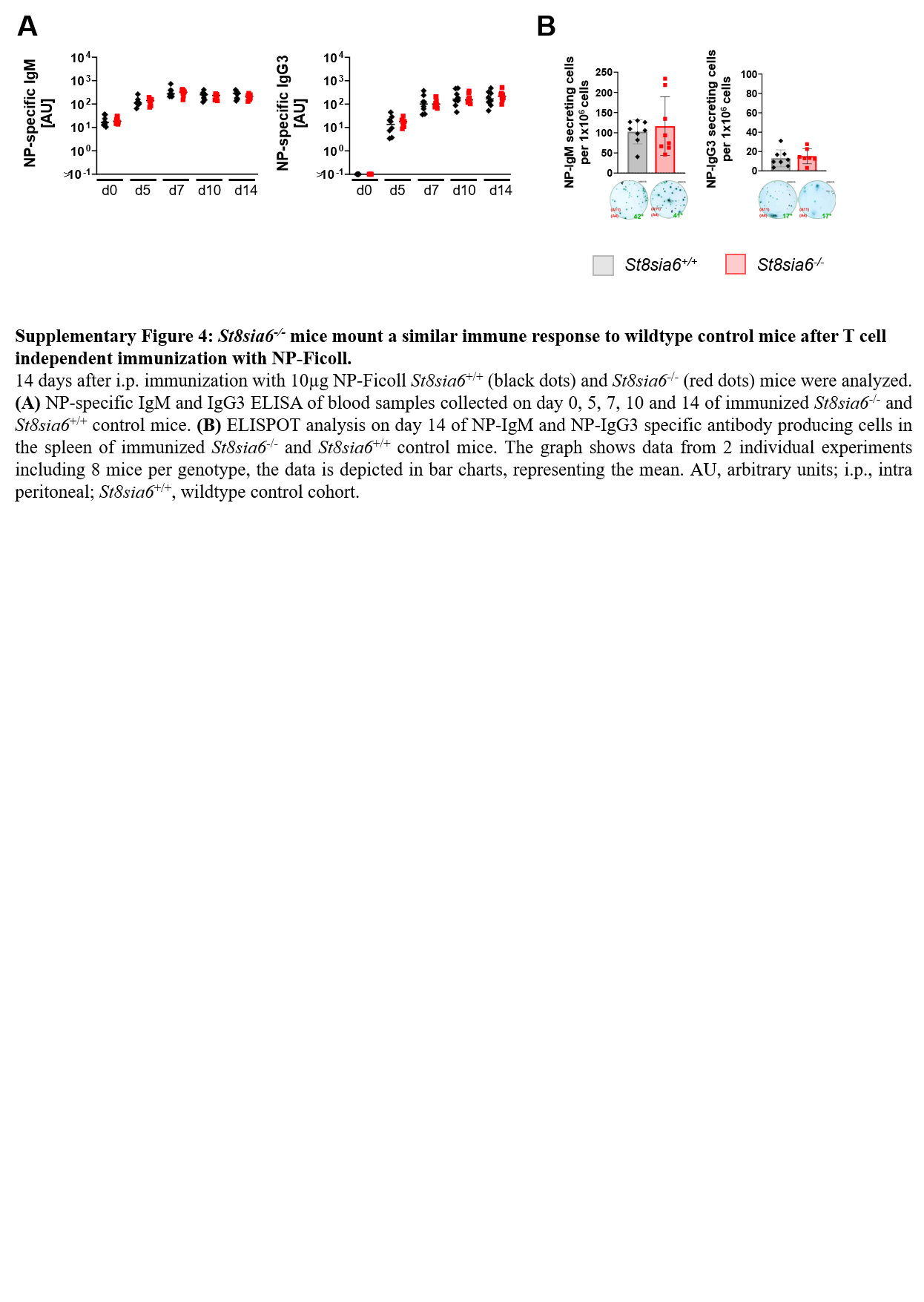

Supplement: Supplementary file 5 [file Image4.tif]

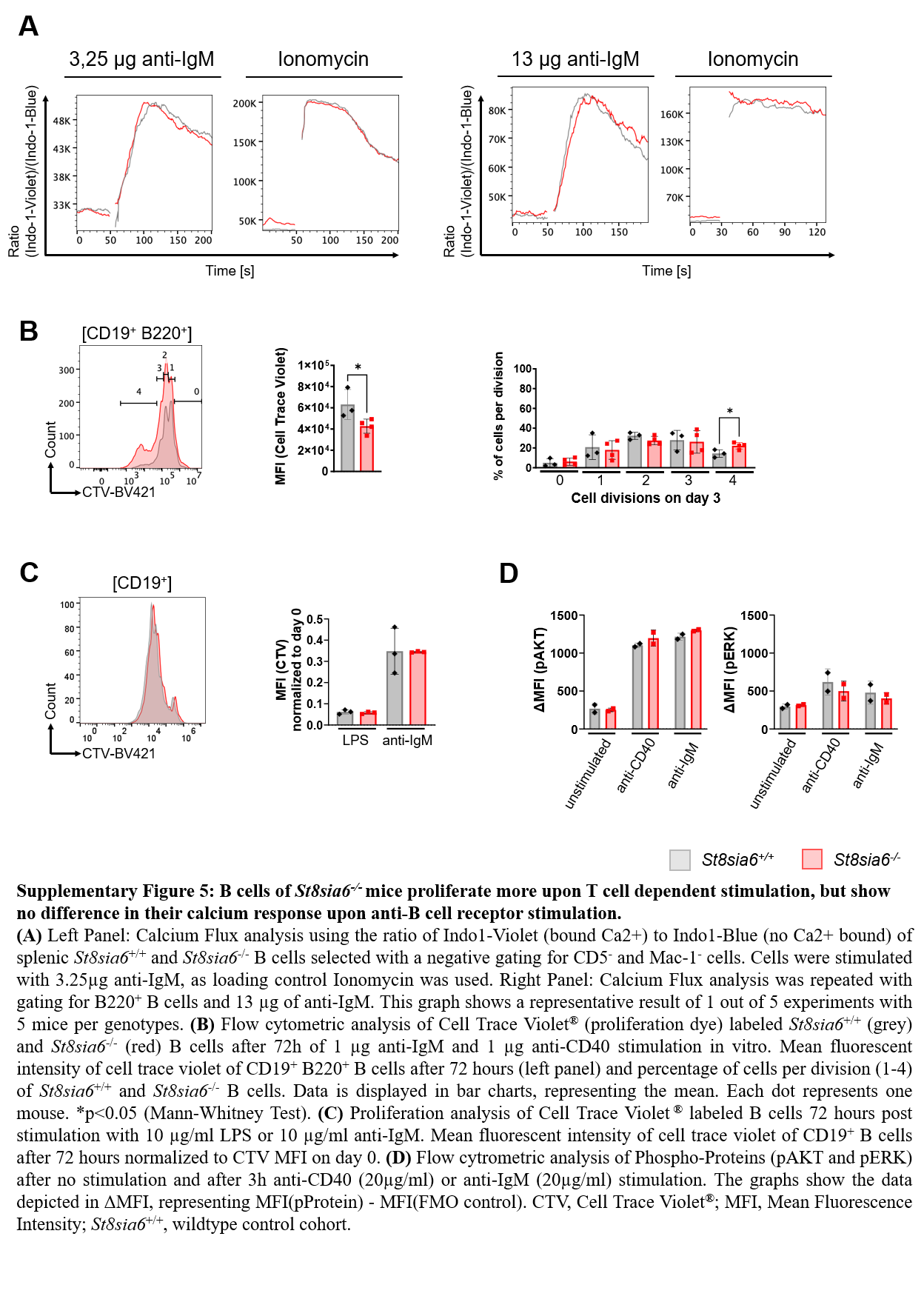

Supplement: Supplementary file 6 [file Image5.tif]
